# HIV subtype-specific gp140-CD4 binding, Temsavir efficacy, and identification of novel adhesion inhibitors against Chinese HIV strains

**DOI:** 10.3389/fimmu.2025.1648546

**Published:** 2025-09-22

**Authors:** Tianyang Liu, Xiaowen Li, Xiaolin Yang, Shengjie Zhang, Yao Wang, Siwei Zhang, Lin Cheng, Shanshan Tang, Fuxiang Wang, Yao Zhao, Hongzhou Lu, Lanlan Wei

**Affiliations:** 1 Institute for Hepatology, Shenzhen Third People’s Hospital, The Second Hospital Affiliated to Southern University of Science and Technology, Shenzhen, Guangdong, China; 2 National Clinical Research Center for Infectious Disease, Shenzhen Third People’s Hospital, The Second Hospital Affiliated to Southern University of Science and Technology, Shenzhen, China; 3 Department of Biology, School of Medicine, Institute for Infectious Disease, Southern University of Science and Technology, Shenzhen, China; 4 Shenzhen Key Laboratory of Pathogen and Immunity, Shenzhen Clinical Research Center for Infectious Disease, Shenzhen, China; 5 State Key Discipline of Infectious Disease, Shenzhen Third People’s Hospital, The Second Hospital Affiliated to Southern University of Science and Technology, Shenzhen, China; 6 Department of Basic Medicine, Jiamusi University, Jiamusi, Heilongjiang, China; 7 Institute for Infectious Disease, The Affiliated Hospital of Southwest Medical University, Luzhou, Sichuan, China; 8 Department of Infectious Diseases, Shenzhen Third People’s Hospital, The Second Hospital Affiliated to Southern University of Science and Technology, Shenzhen, Guangdong, China

**Keywords:** HIV-1, gp140-CD4 binding, Temsavir, CRF subtypes, adhesion inhibitors

## Abstract

**Introduction:**

The HIV epidemic in China is characterized by significant genetic diversity with multiple circulating recombinant forms (CRFs). The gp120-CD4 interaction, essential for viral cellular entry, exhibits subtype-dependent structural variations that compromise therapeutic efficacy. Although temsavir remains the only FDA-approved adhesion inhibitor, its activity against predominant HIV subtypes in China has not been systematically evaluated.

**Methods:**

HIV subtyping of 472 clinical samples identified five major strains (B, CRF01_AE, CRF07_BC, CRF08_BC, and CRF55_01B). Recombinant gp140 proteins from these subtypes were expressed, purified, and analyzed via bio-layer interferometry (BLI) to characterize CD4 binding properties. Structural analyses compared hydrogen bonding and interface buried surface areas. Temsavir’s inhibitory efficacy was assessed, and virtual screening of 13,819 compounds combined with BLI validation was performed to identify novel inhibitors.

**Results:**

Subtype B demonstrated the strongest CD4 binding affinity (KD=79 pM), while CRF55_01B showed the weakest binding (KD=8.76 nM). CRF variants exhibited reduced hydrogen bonding and smaller interface buried surface areas, correlating with diminished binding affinity. Temsavir inhibition was subtype-dependent, achieving 35.7% inhibition for subtype B versus <1.3% for CRF01_AE and CRF55_01B, primarily due to steric hindrance induced by the S375H mutation. Five novel inhibitors targeting CRF55_01B were identified with inhibition rates 19%.

**Discussion:**

This study elucidates the molecular mechanisms of HIV-1 adhesion variation and provides specific candidate HIV adhesion inhibitors for prevalent CRF subtypes in China. Subsequent efforts will focus on preclinical validation and structure–activity relationship optimization of these candidates, laying the groundwork for developing personalized therapeutic strategies against region-specific strains.

## Introduction

The Human Immunodeficiency Virus (HIV), characterized by its exceptional genetic plasticity and complex replication cycle, remains a formidable global health challenge (1). As a lentivirus, HIV exhibits a distinctive architecture featuring envelope glycoproteins (gp120/gp41 trimers) critical for host cell engagement (2). The viral life cycle sequentially proceeds through receptor-mediated attachment, membrane fusion, reverse transcription, proviral integration, and culminating in virion assembly and release (3). The initial gp120-CD4 interaction at the host cell surface triggers conformational changes to enable coreceptor binding and fusion pore formation, which constitutes a rate-limiting step in viral entry (4).

HIV’s evolutionary divergence manifests through two principal types (HIV-1/HIV-2) and extensive subtype diversification (5). HIV-1, responsible for 95% of global AIDS cases, comprises four groups (M, N, O, P) with group M subtypes and circulating recombinant forms (CRFs) dominating epidemics (6). HIV exhibits high genetic variability both between and within subtypes, frequently giving rise to new hybrid viruses through genetic recombination (7). In China, prevalent CRFs like CRF01_AE, CRF07_BC, CRF08_BC, and CRF55_01B complicate prevention and treatment (8). As an important glycoprotein on the HIV envelope, gp120 has a complex and variable structure. It recognizes and binds to the CD4 receptor on host cells, and this binding is the first step for viral entry and crucial for replication (9). Differences in gp120’s sequence and structure among subtypes affect its binding to CD4, creating therapeutic challenges for entry inhibitors (10, 11).

Given the key role of gp120-CD4 binding in HIV infection, developing drugs targeting this interaction is a vital therapeutic strategy. These drugs block gp120-CD4 binding to prevent viral entry and control infection (12–14). However, gp120 structural differences among subtypes cause variations in drug efficacy, affecting drug selection and assessment (15, 16). Currently, Fostemsavir (with Temsavir as its active form) is the only FDA-approved HIV adhesion inhibitor, but it has limited efficacy against HIV CRF subtypes (17). Therefore, researching the structure and function of gp120 in different HIV subtypes and its interaction with CD4 is crucial for more effective and precise HIV treatments.

This study addresses subtype-dependent variations in HIV adhesion through structural and computational analyses of prevalent Chinese strains. We quantify gp140-CD4 binding affinities across subtypes, characterize temsavir resistance mechanisms, and identify novel small-molecule inhibitors with enhanced activity against therapy-resistant CRF variants. Our findings provide critical insights for developing geographically optimized entry inhibitors tailored to China’s evolving HIV epidemic.

## Materials and methods

### Search strategy

Systematic literature searches were conducted in PubMed, Medstrade Foreign Language Retrieval Platform, China National Knowledge Infrastructure (CNKI), Wanfang Data, and Chinese Core Journals Database (up to 31 December 2024) using Boolean operators: (“HIV-1” OR “AIDS”) AND (“China” OR “Chinese”) AND (“subtype” OR “genotype”). Articles in English/Chinese were screened for nationwide HIV-1 subtype prevalence data. Single-province studies were excluded to minimize regional bias. Reference lists of systematic reviews were cross-checked to identify additional studies.

### Clinical HIV subtyping

A cohort of 472 HIV-infected individuals diagnosed at Shenzhen Third People’s Hospital (2023-2024) was enrolled. Whole blood samples (10 mL) were processed using a high-purity viral RNA extraction kit (plasma-derived). Nested RT-PCR targeting a 1,062-bp pol gene fragment (HXB2 coordinates 2253-3314) was performed with One-Step RNA PCR reagents, ExTaq polymerase, and subtype-specific primers. Amplicons were sequenced by Dongguan HyLabs virology laboratory. Sequence quality was validated using the LANL HIV Sequence Quality Control Tool (https://www.hiv.lanl.gov/content/sequence/QC/index.html, accessed on 1 July 2023–20 August 2025). Recombinant strains were identified via RIP and jpHMM algorithms in the HIV Database. Phylogenetic analysis (MEGA 11, Kimura 2-parameter model, 1,000 bootstrap replicates) confirmed subtypes. Drug resistance profiles were determined through Stanford HIV Drug Resistance Database submissions.

### Cell lines

HEK293F human embryonic kidney cells (obtained from ATCC, CBP-60437) were maintained at 37°C under 5% CO_2_ in SMM 293-TII Expression Medium (Serum free, complete medium, M293TII, Sino Biological), supplemented with 1-5% SMS 293-SUPI Expression Medium Supplement (M293-SUPI, Sino Biological). TZM-bl cells (a HeLa-derived indicator cell line expressing β-galactosidase and luciferase reporter genes under the control of the HIV promoter) and 293T cells (kindly provided by Prof. Lin Cheng) were cultured in medium supplemented with: 10% fetal bovine serum (FBS; A5669701, GIBCO), 1% penicillin-streptomycin solution (10,000 U/mL; 15140122, GIBCO) and maintained in a humidified incubator at 37°C with 5% CO_2_.

### Plasmids

HIV-1 gp140 sequences (subtypes B, CRF01_AE, CRF07_BC, CRF08_BC, CRF55_01B; Los Alamos HIV Database) were cloned into pcDNA3.1 (V79020, Thermo Fisher) with C-terminal FLAG tags. Human CD4 (UniProt P01730) was cloned with a His_6_ tag. Constructs were transformed into E. coli TOP10 (C404010, Thermo Fisher), and plasmid integrity was confirmed via Sanger sequencing (Genewiz).

### Protein expression and purification

HEK293F cells (2×10^6^ cells/mL) were transfected with 1 μg/mL plasmid using EZ Trans (AC04L092, Life-iLab; DNA:reagent=1:5). Culture supernatants harvested at 120 hr were filtered (0.45 μm), loaded onto Anti-FLAG M2 Affinity Gel (A2220, Sigma-Aldrich), and eluted with 3×FLAG peptide (150 μg/mL, F3165, Sigma-Aldrich). Purified gp140 was concentrated (30 kDa Amicon Ultra, UFC9030, Merck), subjected to size-exclusion chromatography (Superose 6 Increase 10/300 GL, 29091559, Cytiva), and trimer fractions (420–450 kDa) were collected. Purity (>95%) was confirmed by SDS-PAGE (PG610, Epizyme). The PageRuler™ Unstained Protein Ladder (26614, Thermo Scientific) was employed as a molecular weight standard for protein size estimation during SDS-PAGE.

### Small molecule compounds

The HIV attachment inhibitor temsavir (BMS-626529) was purchased from MedChemExpress (HY-15440). The compound was dissolved in dimethyl sulfoxide (DMSO, 276855, Sigma-Aldrich) at a stock concentration of 10 mM, aliquoted, and stored at −80°C until further use. The screened compounds were purchased as prepared solutions from Shanghai Topscience. The specific information is shown in **Supplementary Table 1**.

### BLI analysis

CD4 (10 μg/mL) was immobilized on Ni-NTA biosensors (160016, GatorBio) for 60 s. Association/dissociation kinetics between gp140 (6.25–200 nM) and CD4 were measured at 25°C using a GatorPrime system. For inhibition assays, gp140 (250 nM) was pre-incubated with temsavir (50 μM, HY-15440, MedChemExpress)or candidate compounds (**Supplementary Table S2**). Data were analyzed using Gator One v2.3 (1:1 binding model, global fitting). Inhibition rates were calculated as: *1–[Response(gp140+inhibitor)/Response(gp140)]*.

### HIV infection and drug treatment

HIV pseudovirions were generated by transfecting 293T cells (1×10^6^ cells/well) with 4 μg of full-length infectious HIV plasmid (pNL4–3 luc R-E- plasmid + Env plasmid) (kindly provided by Lin Cheng) using Lipofectamine 2000 transfection reagent (11668030, Thermo Fisher Scientific) in OPTI-MEM reduced-serum medium (31985070, Gibco). Transfected cells were cultured at 37°C for 48 hours. Virus-containing supernatants were filtered through 0.45-μm filters to remove cellular debris and stored at -80°C until use.

For infection, 500 μL of pseudovirus (MOI = 0.25~1) was co-incubated with small-molecule drugs at room temperature for 2 hours, then added to TZM-bl cells (5×10^5^ cells/well) for infection and drug inhibition. After 4 hours, unbound viruses were removed by washing cells five times with phosphate Buffered Saline (PBS) buffer, followed by the addition of fresh medium. Supernatants were harvested for ELISA after 48 hours, and cellular RNA was extracted for HIV nucleic acid detection.

### Cell viability assay

Cell viability was evaluated using the Cell Counting Kit-8 (CCK-8; 100-120, Goonie) according to the manufacturer’s instructions. Briefly, cells were seeded in 96-well plates at a density of 5×10³ cells per well and incubated overnight to allow attachment. Following the respective treatments, 10 μL of CCK-8 reagent was added to each well, and the plates were incubated at 37°C for 2 hours. The absorbance was measured at 450 nm using a microplate reader (BioTek, USA). All experiments were performed in triplicate and repeated at least three times independently. Cell viability was calculated as a percentage relative to the untreated control group.

### ELISA

Using the HIV-1 Gag p24 ELISA Kit (GE0001, Service), prepare standards and reagents according to the manufacturer’s instructions prior to the experiment. Add 100 μL of sample (standard or test sample) and 50 μL of detection antibody to each well sequentially, completing all additions within 15 minutes. Mix well and incubate with shaking for 2 hours at room temperature. Wash the plate 5 times and pat dry, then add 100 μL of detection antibody working solution to all wells and incubate with shaking for 1 hour at room temperature. Wash again 5 times and pat dry, add 90 μL of TMB substrate to each well and incubate for 10–30 minutes at room temperature in the dark. Finally, add 50 μL of stop solution to all wells. Measure the absorbance within 10 minutes using dual wavelengths of 450 nm and 630 nm. The calibrated OD value is obtained by subtracting the 630 nm measurement from the 450 nm measurement.

### RNA extraction and RT-qPCR

Total RNA was isolated from cells using TRIzol Reagent (15596026CN, Thermo Fisher Scientific) according to the manufacturer’s protocol. cDNA was synthesized with the PrimeScript™ RT Reagent Kit (Perfect Real Time) (RR037A, Takara Bio) following the manufacturer’s instructions. HIV-1 RNA levels were quantified by real-time PCR targeting the Nef gene, with the following primers: Forward: 5’-GGTGGGTTTTCCAGTCACAC-3’;​Reverse: 5’-GGGAGTGAATTAGCCCTTCC-3’. PCR amplification was performed using TB Green^®^ Premix Ex Taq™ II (Tli RNaseH Plus) (RR820A, Takara Bio) on a QuantStudio 7 Flex Real-Time PCR System (Viia 7DX, Applied Biosystems). All HIV-1 copy numbers were normalized against endogenous TBP mRNA levels in the same samples, amplified with primers: Forward: 5’-CACGGCACTGATTTTCAGTTCT-3’; Reverse: 5’-TTCTTGCTGCCAGTCTGGACT-3’. Expression alterations were quantified via the 2^−ΔΔCT^ method, with comparisons referenced to negative controls.

### Sequence analysis

The HIV sequences were derived from Chinese samples in the Los Alamos HIV Sequence Database (http://www.hiv.lanl.gov; accessed on 1 July 2023–20 August 2025). Clustraw (https://www.genome.jp/tools-bin/clustalw; accessed on 14 July 2023–20 March 2024) was used for sequence comparison, Esprint 3.0 (https://espript.ibcp.fr/ESPript/ESPript/index.php; accessed on 14 July 2023–20 March 2024) was used for sequence alignment visualization and secondary structure prediction, and Jalview software to export different subtype consensus sequence colors for automatic generation. The Logo plots for HIV were made using the Analyze Align tool at the HIV database and are based on the WebLogo 3 program (https://weblogo.threeplusone.com; accessed on 1 October-28 December 2023) and the HIV-1 database global curated and filtered 2021 alignment published, including 5 HIV-1 gp120 protein sequence per person from 3, 878 individuals in China. The relative height of each letter within individual stack represents the frequency of the indicated amino acid at that position. The numbering of all the Env amino acid sequences is based on the prototypic strain of HIV-1. Use Jaview (https://www.jalview.org; accessed on 11 October-28 December 2023) to compare sequence edits and conduct percentage statistics.

### Molecular dynamics simulation parameters

All molecular dynamics (MD) simulations were performed using the GROMACS 202X software package. The following force fields were employed: AMBER99SB-ILDN for proteins, GAFF for ligands, and TIP3P for water molecules. The system was solvated in a cubic water box with a minimum distance of 1.2 nm between the protein surface and box edges. Simulation parameters included: PME method for long-range electrostatic interactions, and a 1.0 nm cutoff for van der Waals interactions. The protocol consisted of energy minimization, followed by 100 ps NVT ensemble equilibration, and finally 100 ns production simulation under NPT ensemble. Temperature (300 K) and pressure (1 bar) were maintained using V-rescale and Berendsen coupling methods, respectively. Trajectory analysis included: structural stability assessment via Cα-RMSD, and energetic evaluation through MM/PBSA binding free energy calculations (performed every 10 ns) with residue-wise decomposition.

### Structural modeling

The three-dimensional structure of the HIV-1 gp120 monomer was predicted using AlphaFold3 (https://alphafold.com; accessed from 25 June 2024 to 23 August 2024) with the following protocol: The gp120 amino acid sequence (signal peptide removed) obtained from the Los Alamos HIV Sequence Database (http://www.hiv.lanl.gov) served as input; five models were generated using default parameters without template mode, from which the model with the highest confidence (pLDDT > 90) was selected as the initial structure. Subsequent post-processing included energy minimization using the AMBER99SB force field (5000 steps of steepest descent algorithm) and addition of missing hydrogen atoms. The predicted model was then aligned with the experimental structure PDB: 3J70 (HIV-1 subtype B gp120-CD4 complex), with Cα-RMSD analysis confirming the accuracy of core structural regions. Finally, all models underwent stereochemical validation through Ramachandran Plot analysis (https://saves.mbi.ucla.edu/, accessed from 25 June 2024 to 20 December 2024), which demonstrated that >95% of residues occupied allowed regions, ensuring structural reliability.

### Protein-protein docking

CD4 (PDB: 1CDJ) was retrieved from the RCSB Protein Data Bank. GRAMM-X protein-protein docking server (https://gramm.compbio.ku.edu/; accessed on 29 August-22 October 2024) predicted gp120-CD4 complexes using a fast Fourier transform correlation algorithm that evaluates surface complementarity and electrostatic potential matching. Docking parameters: Receptor (CD4) retained crystallographic coordinates; Ligand (gp120) was stripped of water molecules. Grid resolution: 1.0 Å; angular sampling density: 15°; electrostatic weighting: 0.5 (balancing hydrophobic/electrostatic interactions); collision threshold: 50. The top-scoring complex underwent 100 ns MD simulation (GROMACS 2020.6, AMBER99SB-ILDN force field), with the final 20 ns equilibrium trajectory used for structural analysis.

### Protein-ligand docking

Temsavir 3D coordinates were acquired from DrugBank. Protein preparation (water removal, polar hydrogen addition, charge assignment) and flexible residue/rotatable bond definition utilized AutoDock Tools 1.5.6. Semi-flexible docking was executed in AutoDock Vina 1.2.3 (exhaustiveness=25, num_modes=10) within a 30×30×30 Å³ grid box. The optimal pose was selected based on hydrogen bonding and binding free energy. Redocking validation yielded RMSD < 2 Å versus the crystallographic pose.

### Interaction analysis

Protein-protein interfaces were characterized using PDBePISA (http://www.ebi.ac.uk/pdbe/prot_int/pistart.html; accessed on 5 October-30 December 2024) for buried surface area and binding energy quantification. PyMOL (https://PyMOLwiki.org/index.php/InterfaceResidues; accessed on 25 November 2024–23 March 2025) visualized interaction networks, with hydrogen bonds and steric clashes systematically compared across subtypes.

### Result processing and analysis

From GRAMM-X’s top 100 outputs: Hierarchical clustering (PyCluster, RMSD cutoff=5.0 Å) revealed consistent binding modes among the top 10 conformations. The centroid of the largest cluster (ranked #1) was selected as the representative structure. Interface properties (optimal ΔG, contact area) were evaluated via PDBePISA, with docking reliability confirmed by comparison to the known complex (PDB:3J70). This dominant conformation served as the MD starting structure.

### Virtual drug screening

Homology models of gp140 proteins from major HIV-1 subtypes (CRF01_AE, CRF07_BC, CRF08_BC, CRF55_01B, and B) were constructed using SWISS-MODEL, with >96% residues in favored regions of Ramachandran plots. Ligand-binding sites were predicted using MOE’s Site Finder. Protein structures were energy-minimized in Schrödinger Suite (Protein Preparation Wizard) with bond optimization, hydrogen addition, and disulfide/hydrogen bond assignment. MOE and Schrödinger Suite were used under academic licenses from the School of Pharmacy, Fudan University. License configurations follow institutional policies. A chemical library (13,819 compounds) was prepared using LigPrep, generating tautomers, ionization states, and up to 32 conformers per molecule. Molecular docking was performed via Glide’s standard-precision mode, retaining top 50% scoring compounds. Candidates showing≥1.5-order affinity differences across subtypes were prioritized. Protein-ligand interaction fingerprints (PLIF) identified key binding residues for CRF55_01B. Structurally similar compounds were clustered, retaining the highest-affinity representative per cluster.

### Statistical analyses

Statistics were analyzed using GraphPad Prism version 6.01 (GraphPad, San Diego, CA, USA). All data are presented as mean ± SEM. Comparisons between two groups were performed using Student’s t-test. The statistical significance of differences between groups was determined by two-way ANOVA. A p-value below 0.05 was regarded as significant. Experiments were performed in triplicate with biological replicates of n = 3 per condition.

## Results

### Differences in adhesion processes among HIV subtypes

**Figure 1:**
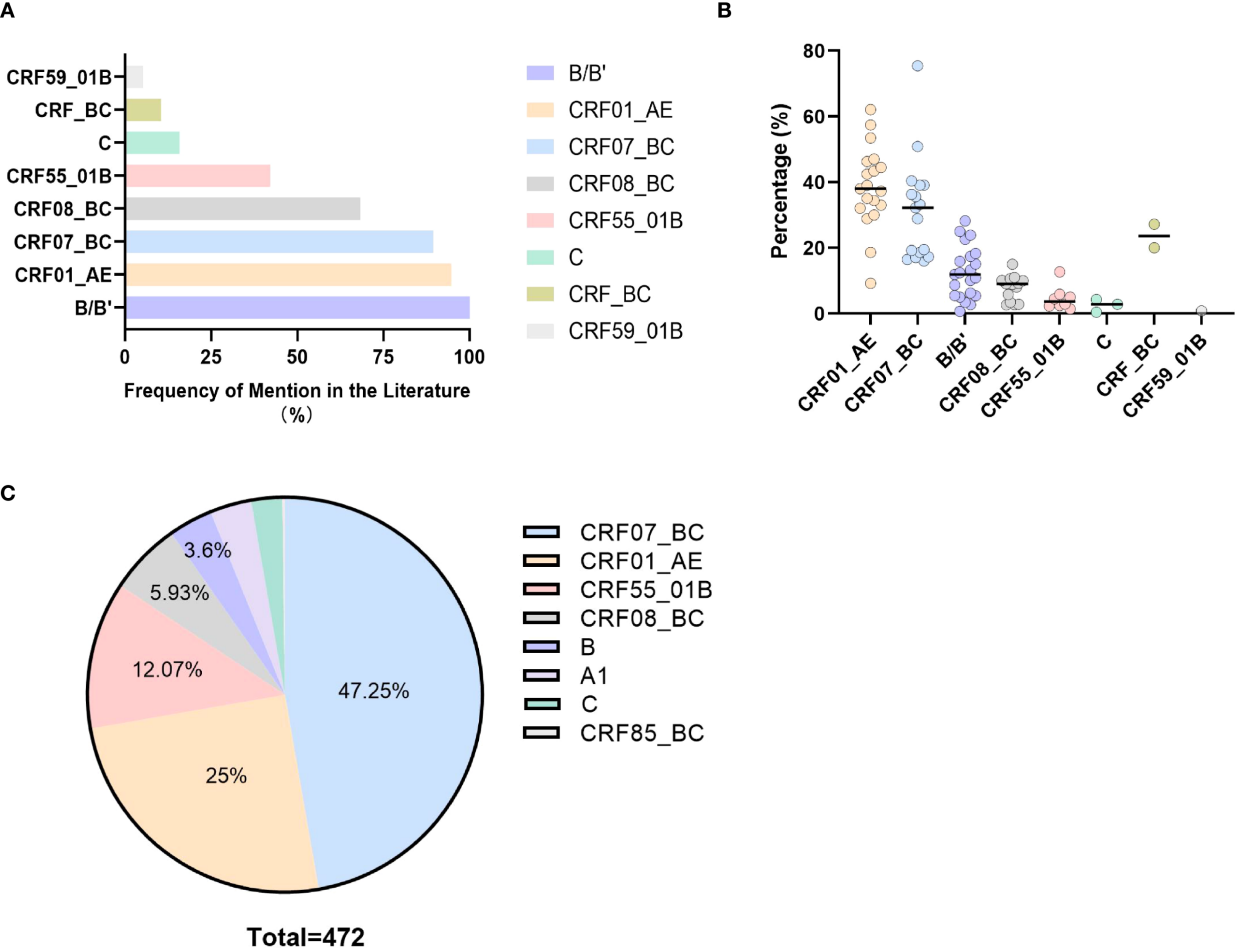
Distribution of HIV-1 subtypes in China based on literature and hospital data. **(A)** Prevalence of the five most common HIV-1 subtypes reported in Chinese literature, **(B)** Proportional distribution of the top five HIV-1 subtypes in Chinese literature, **(C)** HIV-1 subtype distribution among 472 patients in Shenzhen, China, 2024.

To determine the differences in the adhesion processes of different HIV-1 subtypes, search relevant literature on the distribution of HIV-1 in China (981 Chinese and 205 English literatures). To avoid regional differences, we selected only 19 literatures that statistically covered the national distribution. By counting the top 5 HIV-1 subtypes in these literatures, we found that subtypes B (100%, 19/19), CRF01_AE (94.7%, 18/19), CRF07_BC (89.5%, 17/19), CRF08_BC (68.4%, 13/19), and CRF55_01B (42.1%, 8/19) were most frequently mentioned in the Chinese region (**Figure 1A**), and had the highest prevalence rates, which were 11.9%, 38%, 32.2%, 9%, and 3.7% respectively (**Figure 1B**). Clinical validation using 2024 subtyping data from Shenzhen Third People’s Hospital (n=472) demonstrated concordance: CRF07_BC (47.25%, 223/472), CRF01_AE (25%, 118/472), CRF55_01B (12.07%, 57/472), CRF08_BC (5.93%, 28/472), and B (3.6%, 17/472) (**Figure 1C**).

**Figure 2:**
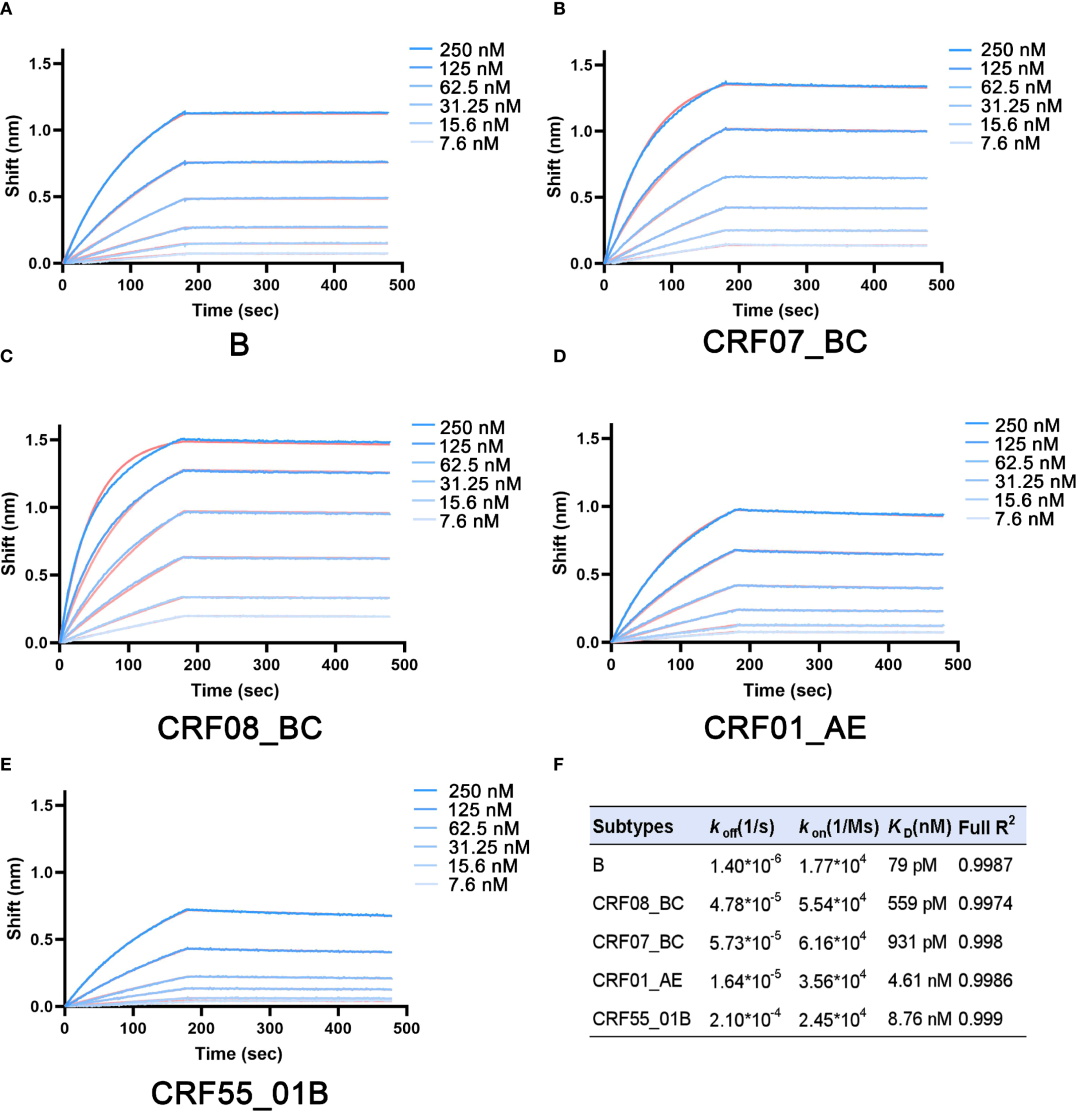
Differences in the binding ability between gp140 and CD4 among different HIV subtypes. **(A)** Subtype B, **(B)** Subtype CRF07_BC, **(C)** Subtype CRF08_BC, **(D)** Subtype CRF01_AE, **(E)** Subtype CRF55_01B, **(F)** Summary of the binding kinetics parameters between gp140 and CD4. *k*_on_: Association Rate Constant; *k*_off_: Dissociation Rate Constant; *K*_D_: Equilibrium Dissociation Constant.

Research shows that fully glycosylated secreted gp140 expressed in mammalian cells can better mimic the envelope protein structure of native HIV conformation. Thus, we purified gp140 (Flag-tagged) from 5 HIV subtypes (B, CRF01_AE, CRF07_BC, CRF08_BC, and CRF55_01B) and human CD4 protein (His-tagged) (**Supplementary Figures 1**, **2**). Bio-layer interferometry (BLI) was used to detect the binding affinity between gp140 at different concentrations and CD4. From the sensorgram, during the binding phase, as the concentration of the ligand gp140 increased, the signal rose rapidly in a concentration-dependent manner. The signal reached a relatively stable state after approximately 200 seconds. Data analysis yielded the equilibrium dissociation constants (*K*_D_) of gp140 proteins from 5 HIV-1 subtypes and the CD4 protein. HIV-1 subtype B displayed the lowest *K*_D_ value (79 pM), indicating the strongest binding affinity. *K*_D_ values increased for CRF07_BC (559 pM) and CRF08_BC (931 pM), while CRF01_AE (4.61 nM) and CRF55_01B (8.76 nM) showed considerably higher *K*_D_ values, representing weaker binding (**Figure 2**).

### The differential mechanisms of the adhesion process of different HIV-1 subtypes

To investigate the mechanisms underlying the differences in the adhesion processes among different HIV subtypes, we conducted a comparative analysis of the gp120 sequences of the five selected HIV-1 subtypes and found numerous differences (**Supplementary Figure 3**). Subsequently, we use Alphafold2 to predict the structures of the gp120 proteins of the five HIV subtypes. Then, these structures were compared with the known HIV-1 B subtype structure in the literature and analyzed in terms of charge distribution, buried area, binding free energy, and hydrogen bond distribution.

The analysis of charge distribution indicated that the binding interface between the CD4 and gp120 proteins was mostly in a region of positive charge distribution, while the corresponding site of gp120 was mostly negatively charged, facilitating their binding. However, no significant differences were observed among different subtypes (**Supplementary Figure 4**). By docking the gp120 proteins of different HIV-1 subtypes with the CD4 protein and analyzing the buried area at the binding site and the binding free energy, we found that subtype B, which had the strongest binding ability, had the largest buried area (1153.6 Å²). The binding free energy results showed that the binding free energy between the gp120 of subtype B and the CD4 protein was -5.6 kcal/mol, indicating a certain degree of binding stability. The buried areas and binding free energies of CRF07_BC and CRF08_BC subtypes were the next, while those of CRF01_AE and CRF55_01B had smaller buried areas and the lowest absolute values of binding free energy (**Supplementary Figure 5**, **Table 1**).

**Table 1:** Buried surface area and dynamic simulation of CD4 binding to different HIV gp120 subtypes.

Subtypes	B	CRF07_BC	CRF08_BC	CRF01_AE	CRF55_01B
Interface area/Å^2^	1153.6	899.3	876.4	808.6	847.3
ΔiG/kcal/mol	-5.6	-2.7	-3.4	-1.6	-0.8

**Figure 3:**
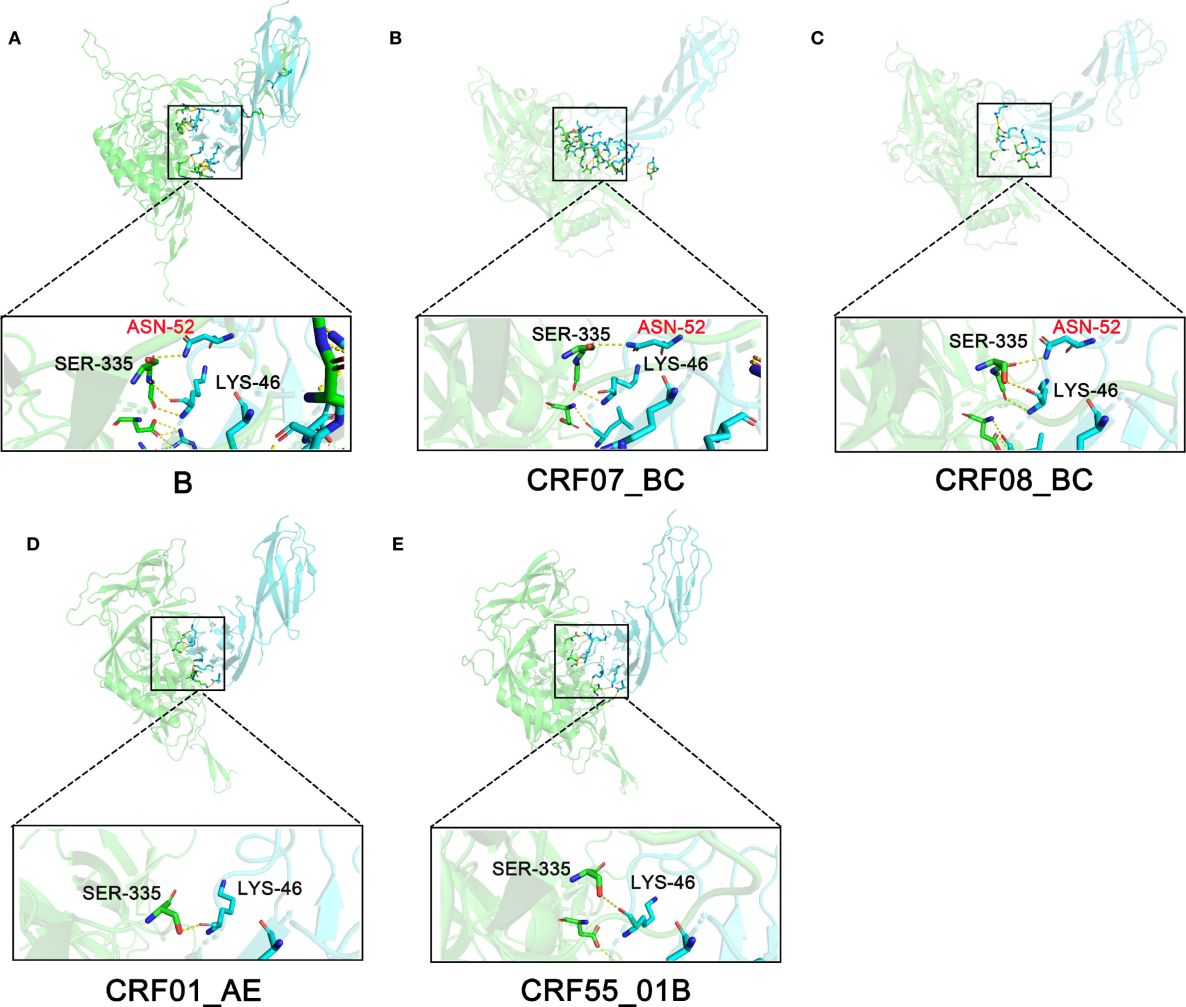
Hydrogen bonds formed between CD4 and gp120 across different HIV subtypes. **(A)** Subtype B, **(B)** Subtype CRF07_BC, **(C)** Subtype CRF08_BC, **(D)** Subtype CRF01_AE, **(E)** Subtype CRF55_01B. CD4 and gp120 are shown in cartoon representation and colored in bule and green, respectively.

Next, we analyzed the hydrogen bonds at the binding interface between gp120 of different HIV-1 subtypes and CD4. We found that there is a more extensive hydrogen-bond network between the gp120 of subtype B and the CD4 protein (**Supplementary Table 2**). Moreover, we discovered that in the gp120 of subtypes B, CRF07_BC, and CRF08_BC, hydrogen bonds are formed between the backbone amide of Ser335 and the side chain of Asn52 of CD4, as well as between the hydroxyl group of Ser335 and the carbonyl oxygen of Lys46. In contrast, CRF01_AE and CRF55_01B, which exhibited poor binding abilities, failed to form hydrogen bonds at this position (**Figure 3**). Through analysis, we believe that the differences in the adhesion processes of different HIV subtypes may be attributed to sequence mutations in the gp120 proteins of different HIV-1 subtypes during evolution. These mutations lead to changes in the buried area at the binding interface with the CD4 protein and the hydrogen bonds at the interaction interface, consequently resulting in differences in binding free energy.

### Temsavir poorly inhibits the adhesion of common HIV subtypes in China

**Figure 4:**
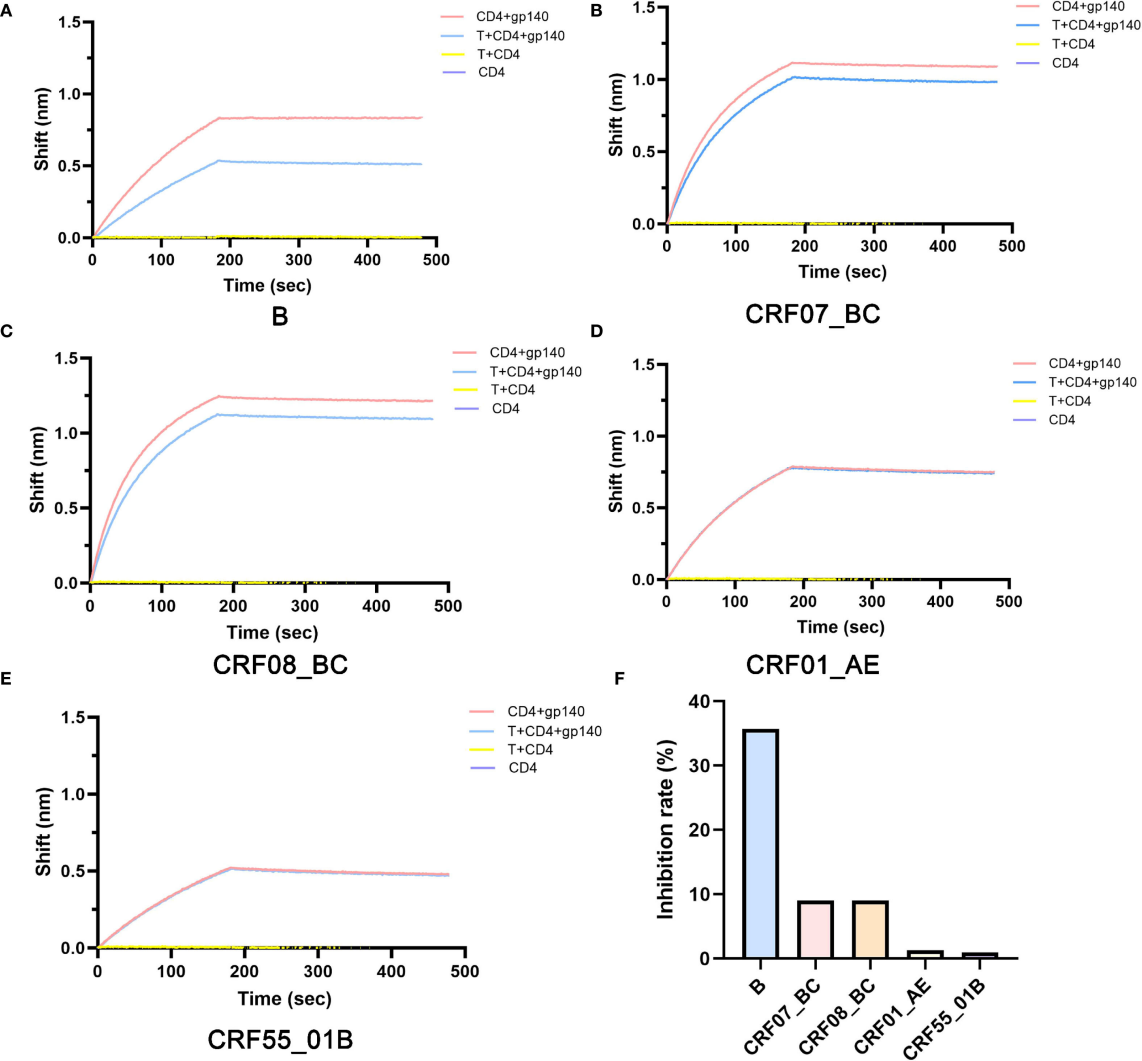
Detection of the differences in the inhibition of gp140-CD4 binding of different HIV subtypes by Temsavir using BLI method. **(A)** Subtype B, **(B)** Subtype CRF07_BC, **(C)** Subtype CRF08_BC, **(D)** Subtype CRF01_AE, **(E)** Subtype CRF55_01B, **(F)** Inhibition rate of Temsavir on gp140-CD4 binding across different HIV subtypes. T:Temsavir.

Subsequently, we used BLI to detect the inhibitory effect of Temsavir, the active precursor form of fostemsavir, the only FDA-approved HIV adhesion inhibitor, on the adhesion processes of different HIV subtypes. The results showed significant differences in the inhibitory effect of Temsavir on the adhesion processes of different HIV subtypes (**Figures 4A–E**). The highest inhibition rate was observed for subtype B (35.7%), followed by CRF07_BC (9%) and CRF08_BC (9%), while the inhibitory effects on CRF01_AE (1.27%) and CRF55_01B (0.96%) were the poorest (**Figures 4F**).

**Figure 5:**
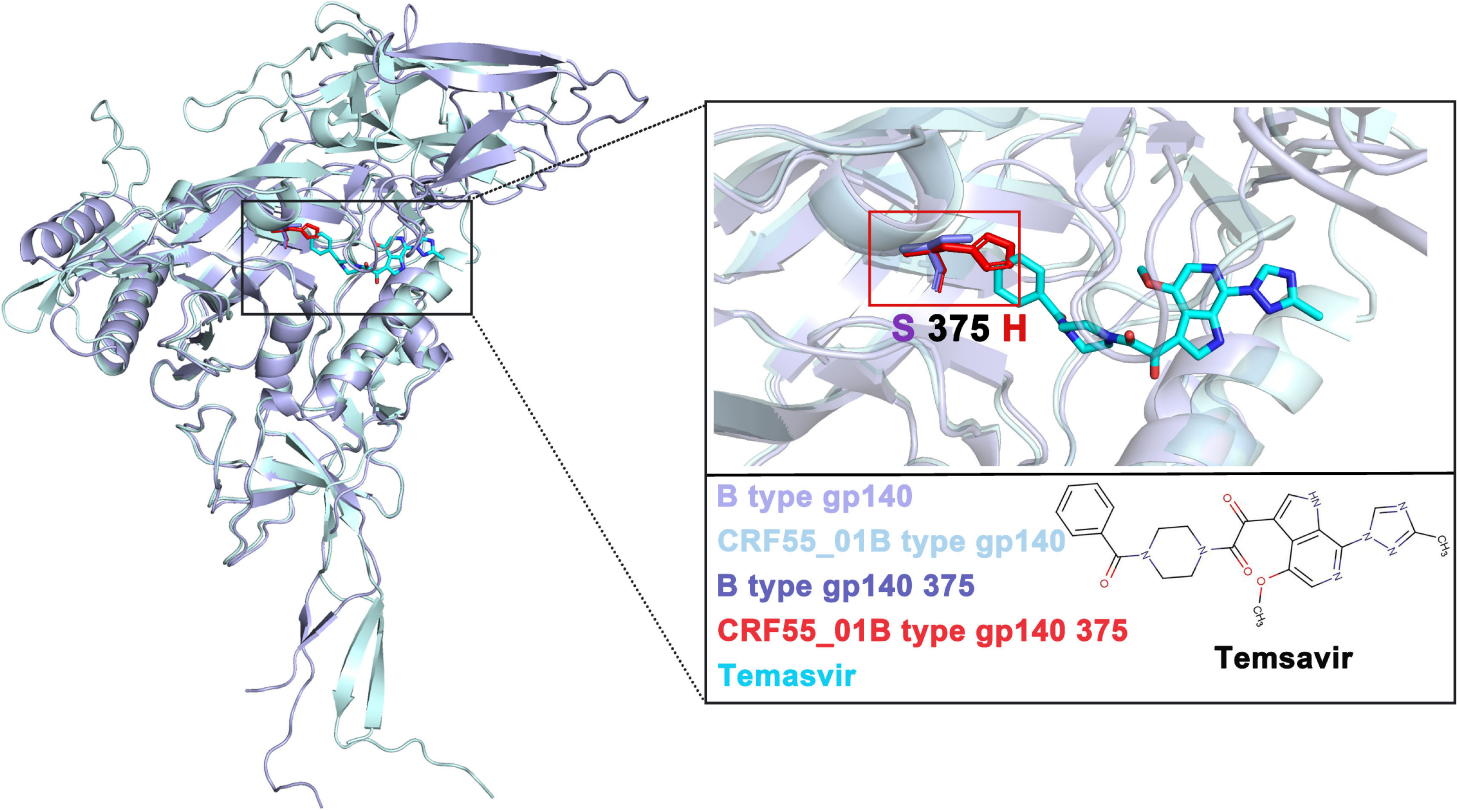
Molecular docking analysis of gp120 of CRF 55_01B and subtype B HIV and the small molecule drug Temsavir.

Through small-molecule docking studies of gp120 and Temsavir, we discovered that CRF01_AE and CRF55_01B failed to successfully dock with Temsavir (**Supplementary Figure 6**). To further explore the underlying reasons, we compared the sequence variations at relevant sites involved in Temsavir’s function (**Supplementary Figure 7A**) and conducted an in-depth analysis of the variation at position 375 in 3,878 sequences from China in the HIV database. The findings revealed that in the gp120 of CRF01_AE and CRF55_01B subtypes, the serine at position 375 mutated to histidine. This mutation generated substantial steric hindrance, which impeded the binding to Temsavir (**Supplementary Figures 7B–F**). This change was highly likely the key factor leading to the failure of CRF01_AE and CRF55_01B subtypes to dock with Temsavir successfully and, consequently, the lack of a significant inhibitory effect (**Figure 5**).

**Figure 6:**
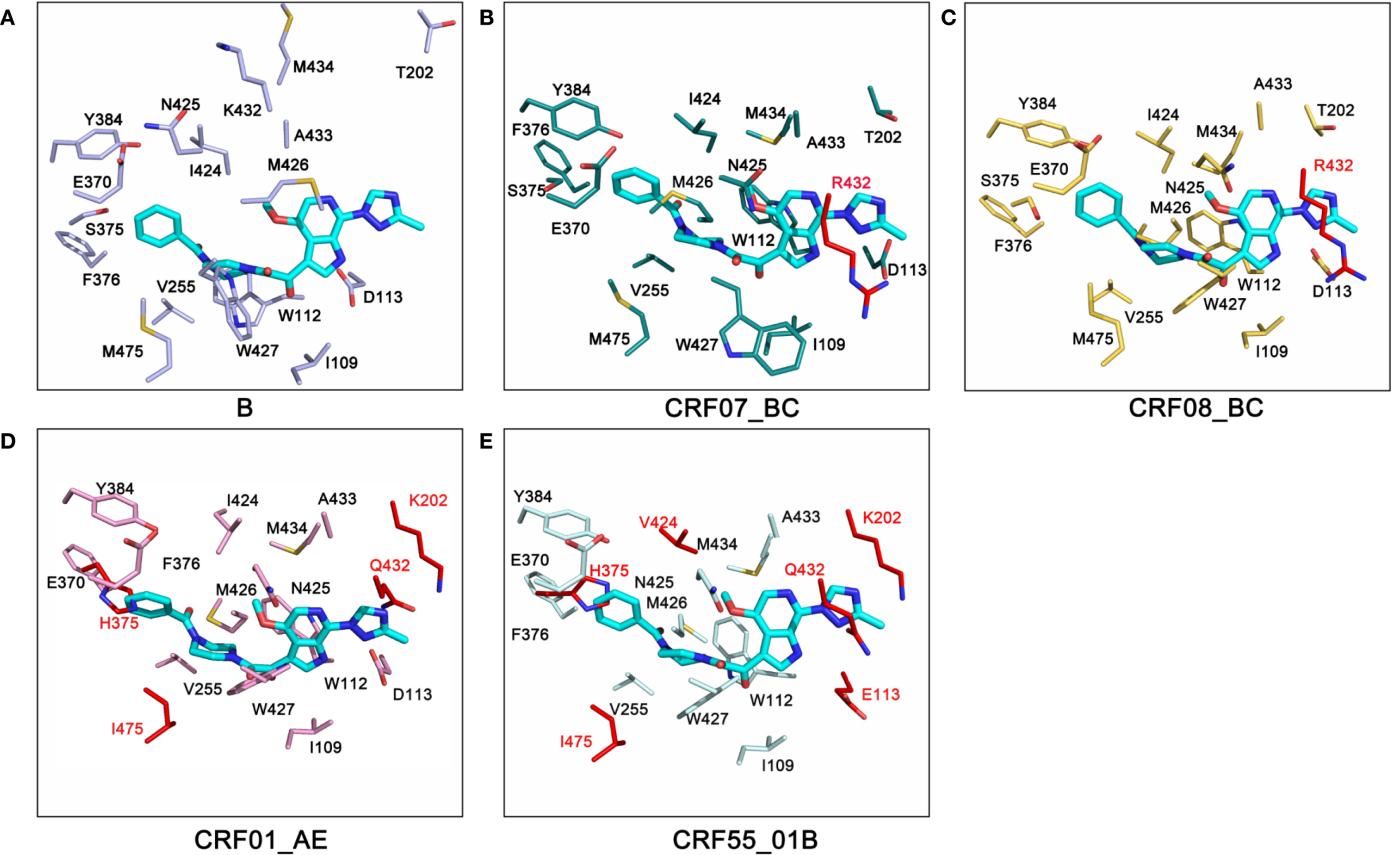
Comparison of residues within 4Å of gp120 and temsavir across different HIV subtypes. **(A)** Subtype B, **(B)** Subtype CRF07_BC, **(C)** Subtype CRF08_BC, **(D)** Subtype CRF01_AE, **(E)** Subtype CRF55_01B. The blue compound structure represents Temsavir.

Next, we carried out a detailed analysis of the residues within a 4 Å distance between gp120 and Temsavir in five HIV subtypes. The results showed that, from the perspective of the overall spatial conformation, the distance between residues in subtype B was relatively large. Its binding pocket space was more expansive compared to other subtypes, and it was also farther from the spatial position of the small-molecule inhibitor. This unique spatial structure might be more conducive to the entry and binding of Temsavir. When comparing the residue mutations among subtypes, we found that in CRF07_BC and CRF08_BC subtypes, a mutation from lysine (K) to arginine (R) occurred at position 432. In CRF01_AE, mutations were present at sites T202K, S375H, K432Q, and M475I, while in CRF55_01B, mutations occurred at sites D113E, T202K, S375H, I424V, K432Q, and M475I. These mutations were likely important factors contributing to the differences in inhibitory effects among subtypes (**Figure 6**).

### Screening of HIV adhesion inhibitors effective against common subtypes in China

Given the limited efficacy of FDA-approved HIV adhesion inhibitors against common CRF subtypes in China, we performed molecular docking using Schrödinger software between 13,819 compounds and the gp140 protein of the CRF55_01B subtype (Standard Precision mode). After energy optimization and retaining the top 50% scoring molecules in each iteration, the compounds were subsequently docked against gp140 proteins from four other subtypes. Compounds exhibiting binding energy differences exceeding 1.5 orders of magnitude were selected (**Supplementary Figures 8**, **9**). From these, 453 compounds with binding affinities below -6 kcal/mol were identified, among which 53 showed consistent differences of 1.5 orders of magnitude across all five subtypes, and 400 met this criterion in at least two subtypes.

Analysis using PLIF revealed that the 53 consensus differential compounds involved 14 residues and formed 31 interaction fingerprints, with frequent interactions observed at Lys119 and Asp208. In contrast, the 400 partial differential compounds engaged 24 residues and yielded 69 interaction fingerprints (**Supplementary Figure 10**). Under a 70% similarity threshold, the compounds were clustered into 252 groups, with the highest-affinity compound retained from each group. Following visual inspection of binding conformations, 22 of the 53 consensus differential compounds and 119 of the 400 partial differential compounds were retained.

**Figure 7:**
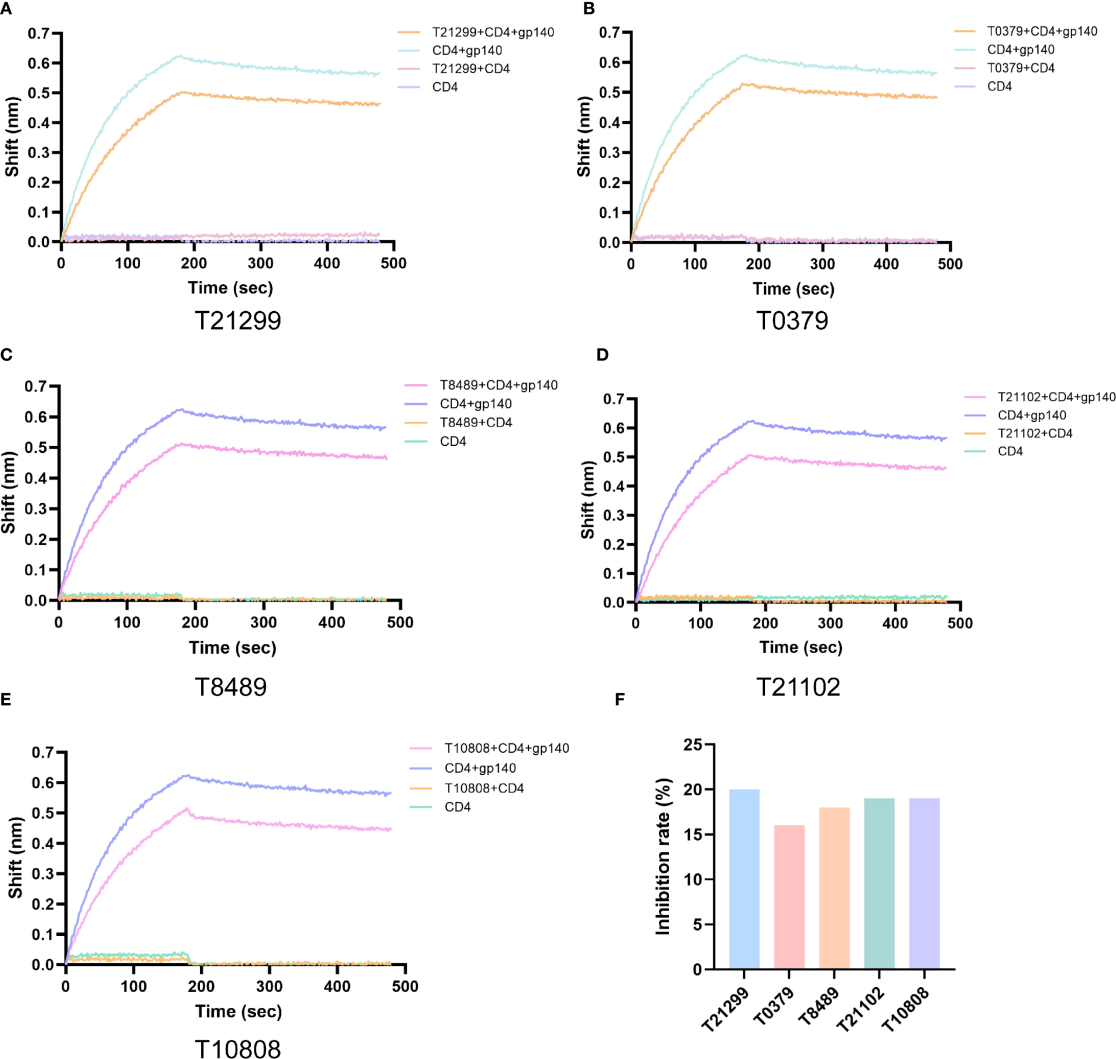
Detection of inhibitory effects of candidate compounds on HIV gp140-CD4 binding. BLI Analysis of Compounds **(A)** T21299, **(B)** T0379, **(C)** T8489, **(D)** T21102, and **(E)** T10808 inhibiting gp140-CD4 binding in CRF55_01B Subtype. **(F)** Inhibition Rates of Compounds on HIV Adhesion Process.

To validate the screening results, the top 10 compounds were ranked based on their Glide scores (see **Supplementary Table 1**). The ability of these compounds to inhibit the interaction between CRF55_01B gp140 and CD4 was evaluated using bio-layer interferometry (BLI) (**Figure 7**, **Supplementary Figure 11**). The results indicated that five compounds exhibited significant inhibitory activity, including two FDA-approved drugs. Notably, T21299 and T10808 showed inhibition rates of 20% and 19%, respectively (**Figure 7**). Subsequently, the cytotoxicity of the compounds toward TZM-bl cells was assessed using the CCK-8 assay. The results indicated that none of the five compounds exhibited significant cytotoxicity after 48 hours of treatment, except for T21102 and T10808 at 100 μM, which showed evident toxicity (**Supplementary Figure 12A**). A non-cytotoxic concentration (10 μM) of each compound was selected for further antiviral evaluation. The compounds were pre-incubated with HIV pseudovirus for 2 hours before inoculation into TZM-bl cells. After 4 hours, the culture medium was replaced. At 48 hours post-infection, ELISA and qRT-PCR were performed to evaluate antiviral activity. The results demonstrated that both T21299 and T8489 exhibited strong anti-HIV activity (**Supplementary Figures 12B, C**). Furthermore, molecular docking was carried out between these five highly effective small molecules and gp140 proteins derived from various HIV subtypes to delineate their binding sites (**Supplementary Figures 13**-**17**, **Supplementary Table 3**).

## Discussion

Human Immunodeficiency Virus (HIV) is a highly mutagenic retrovirus posing a significant threat to global public health, with multiple circulating subtypes (5). Three molecular epidemiological surveys in China revealed changes in the composition of HIV-1 subtypes, with initial B, B’, and C subtypes gradually being replaced by CRF07_BC, CRF01_AE, and CRF08_BC (18, 19). After 2007, CRF01_AE emerged as the predominant strain (8). Following the discovery of the CRF55_01B subtype in Shenzhen, Guangdong, in 2012, its infection rate rapidly increased (20). This study analyzed the distribution of HIV-1 subtypes in China by reviewing previous research and collecting hospital-based sequencing data. The top five subtypes identified were CRF01_AE (38%), CRF07_BC (32.2%), B (11.9%), CRF08_BC (9%), and CRF55_01B (3.7%) (**Figure 1**).

Studies have shown that patients with different HIV subtypes exhibit differences in disease progression and prognosis. A study in South Africa demonstrated variations in the weekly decline rate of CD4+ T cells and disease progression among patients with different subtypes, with subtype D showing a higher CD4+ T cell decline rate and more rapid disease progression (21). Membrane adhesion, specifically the binding of the membrane protein gp120 to the host surface receptor CD4, is a crucial step in HIV infection (22). Considering gp140’s good immunogenicity and its ability to maintain a trimeric form during *in vitro* purification (23, 24), we purified gp140 protein and found significant differences in its binding ability to CD4 among different HIV subtypes (**Figure 2**). The B subtype exhibited the strongest binding ability (79 pM), while CRF55_01B showed the weakest (8.76 nM). Further research revealed that gp140 sequence mutations play a key role in evolution, altering the buried surface area at the binding interface with CD4, the number of hydrogen bonds in the interaction interface, and resulting in changes in binding free energy (**Table 1**). The B subtype’s strong binding ability is attributed to its largest buried surface area and highest number of hydrogen bonds at the CD4 binding interface, with binding free energy data also indicating a more stable and tight connection. CRF07_BC and CRF08_BC subtypes have similar sequences and binding abilities. CRF55_01B, a recombinant subtype of CRF01_AE and B, showed a binding ability similar to CRF01_AE and was relatively weak, as indicated by BLI detection. This may be due to the fewer hydrogen bonds between gp140 and CD4 in these two subtypes, preventing the formation of hydrogen bonds with aspartic acid at position 52 of CD4, resulting in a smaller buried surface area and a lower absolute value of binding free energy. However, while BLI showed weaker binding ability for CRF55_01B, its absolute binding free energy was higher than that of CRF01_AE in structural analysis, possibly due to errors between software-simulated structures and real-world conditions.

In this study, we used AlphaFold2 (25) to predict the tertiary structure of the HIV gp120 protein. Although the core structure (e.g., the CD4 binding site) is known to be highly conserved across different strains (26), AlphaFold2 cannot simulate the glycosylation modifications on the protein surface (which affect antibody binding and CD4 binding kinetics) (27) or the dynamic features of its native trimeric conformation (28). Therefore, the mechanisms underlying the differences in adhesion among various HIV subtypes still require further experimental validation.

Despite relatively mature HIV drug treatment strategies, drug resistance and toxicity remain issues (29, 30). The HIV adhesion process is a key step with a clear target during infection, acting extracellularly without directly interfering with normal intracellular physiological processes, thus offering better safety and tolerability (31). Currently, fostemsavir is the only HIV adhesion inhibitor approved by the FDA, with Temsavir being its precursor active form. Studies have shown variations in the efficacy of Temsavir (BMS-663068) against different HIV subtypes (B, C, D, CRF01_AE, and CRF02_AG), with poorer effects against non-B subtypes (15). Research by Pazgier et al. indicated resistance of the CRF01_AE subtype to Temsavir, primarily due to its Env having histidine at position 375, whose larger side chain directly interferes with temsavir binding to the Phe43 cavity (17). Moreover, six residues (H/Q61, Q105, V108, N474, I475, and K476) in the inner domains 1, 2, and 3 of gp120 coevolve with His375, reshaping the Phe43 cavity and CD4 binding site, altering the formation, stability, and binding conformation of the temsavir binding pocket, making it difficult for temsavir to effectively bind to Env and participating synergistically in the drug resistance process (4, 32, 33).

In our study, Temsavir exhibited the best inhibitory effect on the B subtype (35.7%) and almost no effect on CRF01_AE (1.27%), consistent with previous research. Additionally, we found resistance in the CRF55_01B subtype (0.96%), while its inhibitory effect on the more prevalent CRF07_BC and CRF08_BC subtypes in China was only 9% (**Figure 4**). Mechanism studies revealed that the CRF01_AE and CRF55_01B subtypes cannot bind to Temsavir due to steric hindrance caused by the mutation of serine at position 375 of gp140 to histidine, aligning with previous research (**Figure 5**). Further analysis suggested that the mechanism may be related to mutations at key sites. In terms of overall spatial conformation, compared to other subtypes, the B subtype has a relatively larger distance between gp140 and residues within 4 Å of the small molecule inhibitor Temsavir, potentially allowing easier access and more stable binding. In contrast, the amino acid at position 432 of CRF07_BC and CRF08_BC subtypes mutates from lysine (K) to arginine (R). Arginine has a longer and more complex side chain, resulting in greater steric hindrance. When lysine mutates to arginine, the steric hindrance may prevent small molecule drugs from approaching the binding site, hindering the binding process (34). Furthermore, arginine’s polar guanidino group is more hydrophilic, while lysine’s linear structure favors hydrophobic interactions with non-polar regions (35, 36), leading to stronger hydrophobic interactions between lysine and small molecule drugs. This may explain why Temsavir has a poorer inhibitory effect on CRF07_BC and CRF08_BC compared to the B subtype. The mutation sites for CRF01_AE are T202K, S375H, K432Q, and M475I, and for CRF55_01B are D113E, T202K, S375H, I424V, K432Q, and M475I (**Figure 6**). For these two subtypes, poor drug inhibitory effects are attributed not only to the reported site 375 but also to differences in residues at site 202, which have been reported to alter the local environment of the pocket, affecting the orientation of the methyltriazole ring of temsavir’s “tail”, thereby adjusting temsavir’s binding conformation and significantly impacting its binding efficacy with gp140 (17).

Current development of HIV adhesion inhibitors primarily targets the B subtype prevalent in European and American countries (37). To develop adhesion inhibitors suitable for the HIV subtypes prevalent in China, we carried out molecular docking of the compounds in the compound library with the tertiary structure of the gp140 protein of the CRF55_01B subtype, and then integrated and analyzed the docking results with the data of other subtypes. Initially, we screened out 10 compounds with the highest binding activity (**Supplementary Table 1**), and used BLI to screen out 5 compounds with good inhibitory effects on the CRF55_01B subtype from them, including two marketed compounds and three unmarketed compounds (**Figure 7**). After docking these 5 candidate compounds with the gp120 of other HIV subtypes, they were all able to form hydrogen bond connections with each subtype. These hydrogen bonds are mostly distributed in the Gln113-Leu121 region and at sites such as Cys200, Arg308, Trp423, and Gln424 (**Supplementary Table 3**). From the perspective of spatial conformation, the screened inhibitors basically competitively bind to the gp120 sites with CD4 (**Supplementary Figures 13**-**17**), while Temsavir binds to the gp120 pocket to cause its allosteric change, thereby inhibiting the binding of gp120. Furthermore, the five selected compounds were validated *in vitro* using TZM-bl cells infected with HIV pseudovirus. Among them, T21299 and T8489 exhibited potent antiviral activity (**Supplementary Figure 12**).

However, the inhibitory efficacy of these compounds against different HIV subtypes has not been elucidated in this study. Moreover, further verification using HIV clinical isolates and *in vivo* animal models is still lacking. Therefore, the precise therapeutic potential and underlying mechanisms of action require further extensive investigation. Subsequent efforts will focus on the continued optimization, modification, and functional validation of these compounds.

## Conclusions

This study deciphers the structural determinants of HIV subtype-dependent adhesion dynamics in China, demonstrating that gp140 sequence polymorphisms regulate CD4 engagement efficiency and drive Temsavir resistance. The S375H substitution in CRF01_AE/CRF55_01B and K432R mutation in CRF07/08_BC compromise inhibitor binding through dual mechanisms: steric obstruction of the Phe43 cavity and structural reorganization of the CD4-binding pocket. Furthermore, we identified five novel small-molecule inhibitors showing enhanced suppression (up to 20% increased inhibition) against therapy-resistant strains, establishing a framework for developing precision therapies tailored to China’s predominant CRF subtypes. Future investigations should prioritize preclinical validation of these candidates and structure-activity optimization to counterbalance the evolving HIV genetic landscape, ultimately advancing targeted therapeutic strategies for Chinese HIV patients.

## Data Availability

The datasets presented in this study can be found in online repositories. The names of the repository/repositories and accession number(s) can be found below: the Science Data Bank (https://www.scidb.cn/preview?dataSetId=b229dae9af74436492d281b527b3d517&version=V1) with the assigned DOI: 10.57760/sciencedb.25748.
