# Correction: HIV subtype-specific gp140-CD4 binding, Temsavir efficacy, and identification of novel adhesion inhibitors against Chinese HIV strains

**DOI:** 10.3389/fimmu.2026.1795238

**Published:** 2026-02-04

**Authors:** Tianyang Liu, Xiaowen Li, Xiaolin Yang, Shengjie Zhang, Yao Wang, Siwei Zhang, Lin Cheng, Shanshan Tang, Fuxiang Wang, Yao Zhao, Hongzhou Lu, Lanlan Wei

**Affiliations:** 1 Institute for Hepatology, Shenzhen Third People’s Hospital, The Second Hospital Affiliated to Southern University of Science and Technology, Shenzhen, Guangdong, China; 2 National Clinical Research Center for Infectious Disease, Shenzhen Third People’s Hospital, The Second Hospital Affiliated to Southern University of Science and Technology, Shenzhen, China; 3 Department of Biology, School of Medicine, Institute for Infectious Disease, Southern University of Science and Technology, Shenzhen, China; 4 Shenzhen Key Laboratory of Pathogen and Immunity, Shenzhen Clinical Research Center for Infectious Disease, Shenzhen, China; 5 State Key Discipline of Infectious Disease, Shenzhen Third People’s Hospital, The Second Hospital Affiliated to Southern University of Science and Technology, Shenzhen, China; 6 Department of Basic Medicine, Jiamusi University, Jiamusi, Heilongjiang, China; 7 Institute for Infectious Disease, The Affiliated Hospital of Southwest Medical University, Luzhou, Sichuan, China; 8 Department of Infectious Diseases, Shenzhen Third People’s Hospital, The Second Hospital Affiliated to Southern University of Science and Technology, Shenzhen, Guangdong, China

**Keywords:** HIV-1, gp140-CD4 binding, Temsavir, CRF subtypes, adhesion inhibitors

An incorrect number was provided for the Shenzhen Science and Technology Innovation Program. The correct number is “(KCXFZ20211020163544002)”.

The original version of this article has been updated.

