# Supplementary material for: HIV subtype-specific gp140-CD4 binding, Temsavir efficacy, and identification of novel adhesion inhibitors against Chinese HIV strains
Source: Front Immunol. 2025 Sep 22;16:1648546. doi: 10.3389/fimmu.2025.1648546 (PMC12497853; doi:10.3389/fimmu.2025.1648546)
Supplement: Supplementary file 1 [file DataSheet1.docx]

**Supplementary Figure 1. Purification flow chart of HIV gp140 protein and CD4 protein.**


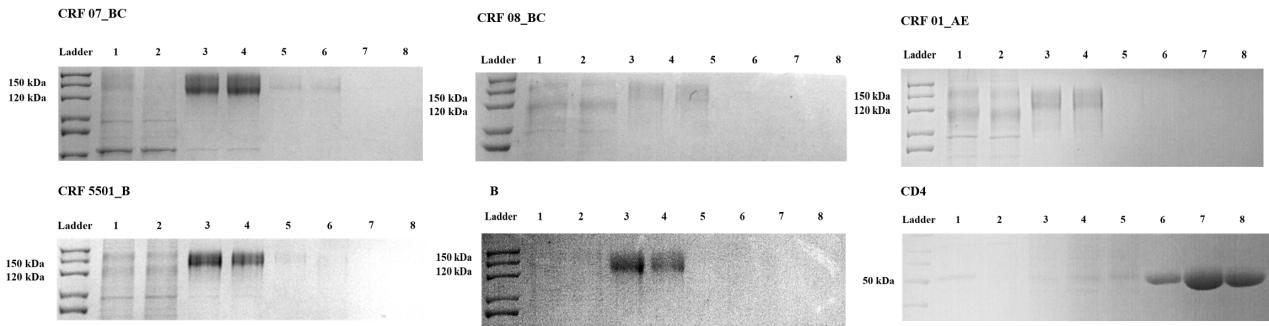


**Supplementary Figure 2. SDS-PAGE analysis of the purification of gp140 and CD4.** (A-E), Lane 1: whole cell fraction (labelled 1); Lane 2: flow through of the cell lysate after application to the column (labelled 2); Lane 3-4: flow through of the imidazole (125 mM) as the elution agent (labelled 3, 4); Laner 5-8: flow through of the imidazole (250 mM) as the elution agent (labelled 5-8). (F), Lane 1: whole cell fraction (labelled 1); Lane 2: flow through of the cell lysate after application to the column (labelled 2); Lane 3-5: flow through of the glycine(pH=3.5) as the elution agent (labelled 3-5); Laner 6-8: flow through of the 3×Flag as the elution agent (labelled 6-8).


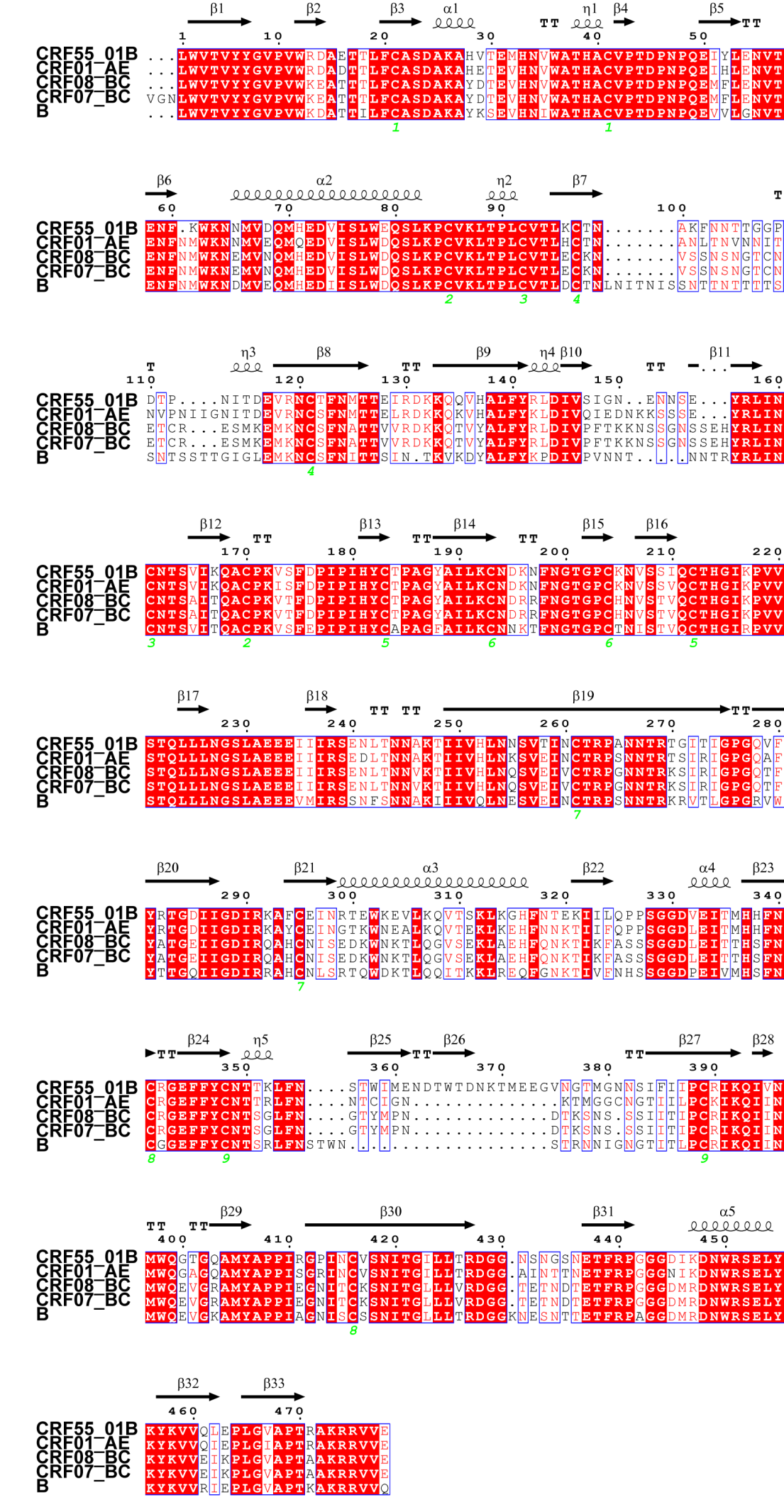


**Supplementary Figure 3. Alignment of gp120 sequences of different HIV subtypes.**


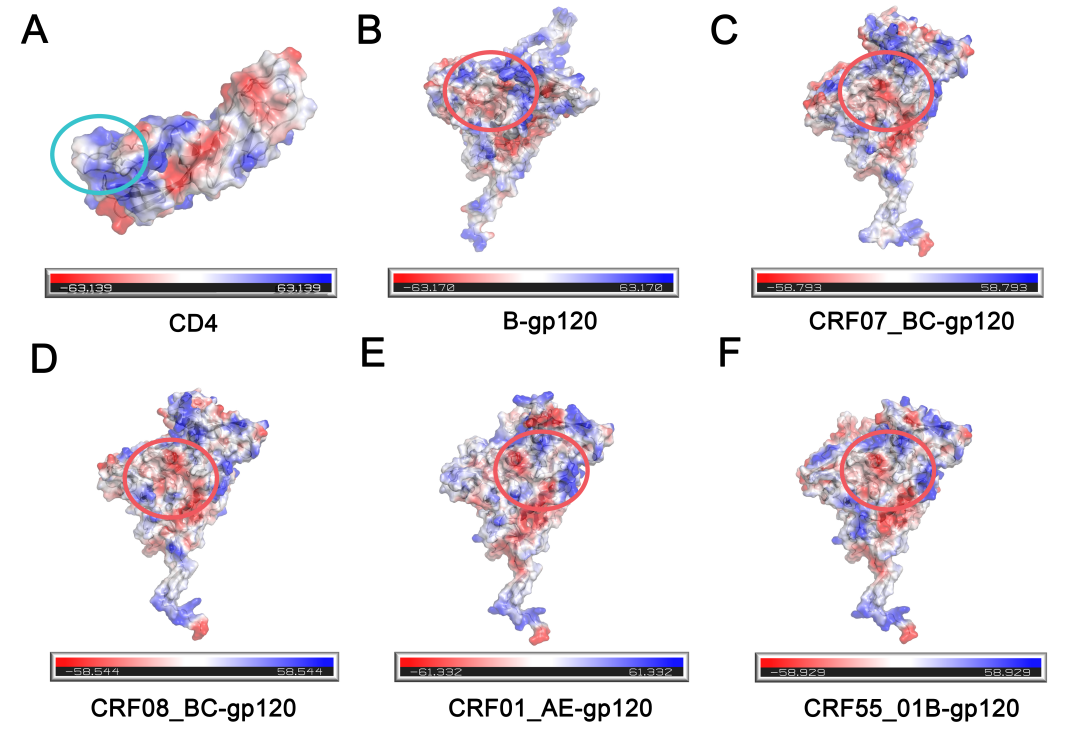


**Supplementary Figure 4. Charge distribution maps of gp120 and CD4 of different subtypes of HIV.** Red represents negative charges and blue represents positive charges.


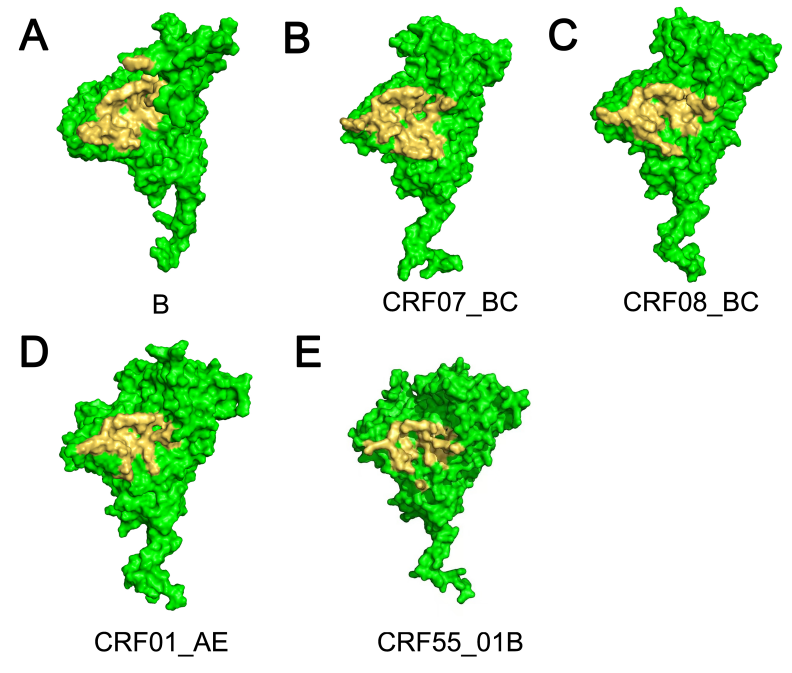


**Supplementary Figure 5. Pymol software was used to analyze the interaction interface between gp120 and CD4 of different subtypes of HIV.** Shown is the surface structure of the protein, with residues located at the interaction interface set in yellow and other residues in green.

**
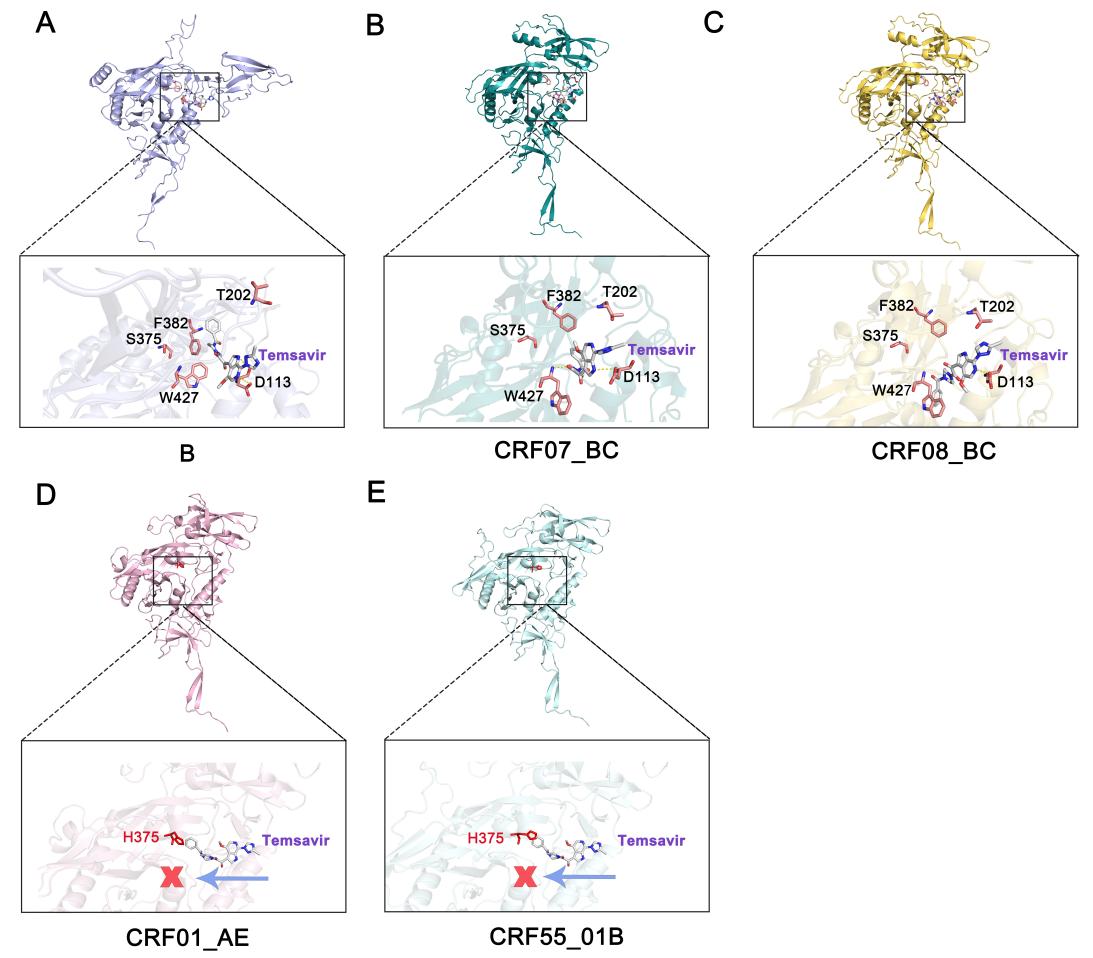
**

**Supplementary Figure 6. Molecular docking analysis of different subtypes HIV gp120 and small molecule drug temsavir.** gp120 is shown as carton, temsavir, and key residues 375S,382F,427W,113D, and 202S in the pocket are shown as sticks.


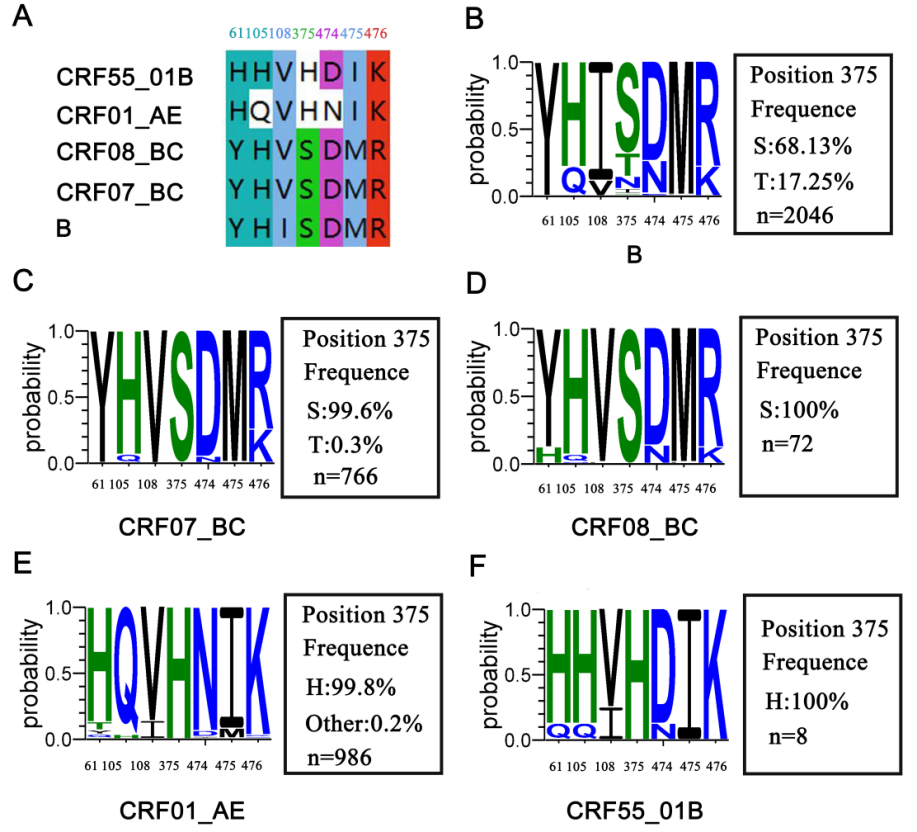


**Supplementary Figure 7. Sequence analysis of gp120 from different HIV subtypes.** (A) Sequence alignment of key residues in gp120. (B-F) Logo depiction of the frequency of each amino acid at positions 61, 105, 108, 375, 474, 475, and 476 within the Phe43 cavity across HIV-1 isolates of Clade B (B), CRE07_BC (C), CRF08_BC (D), CRF01_AE (E), and CRF55_01B (F).The letter height indicates their frequencies in respective clades.


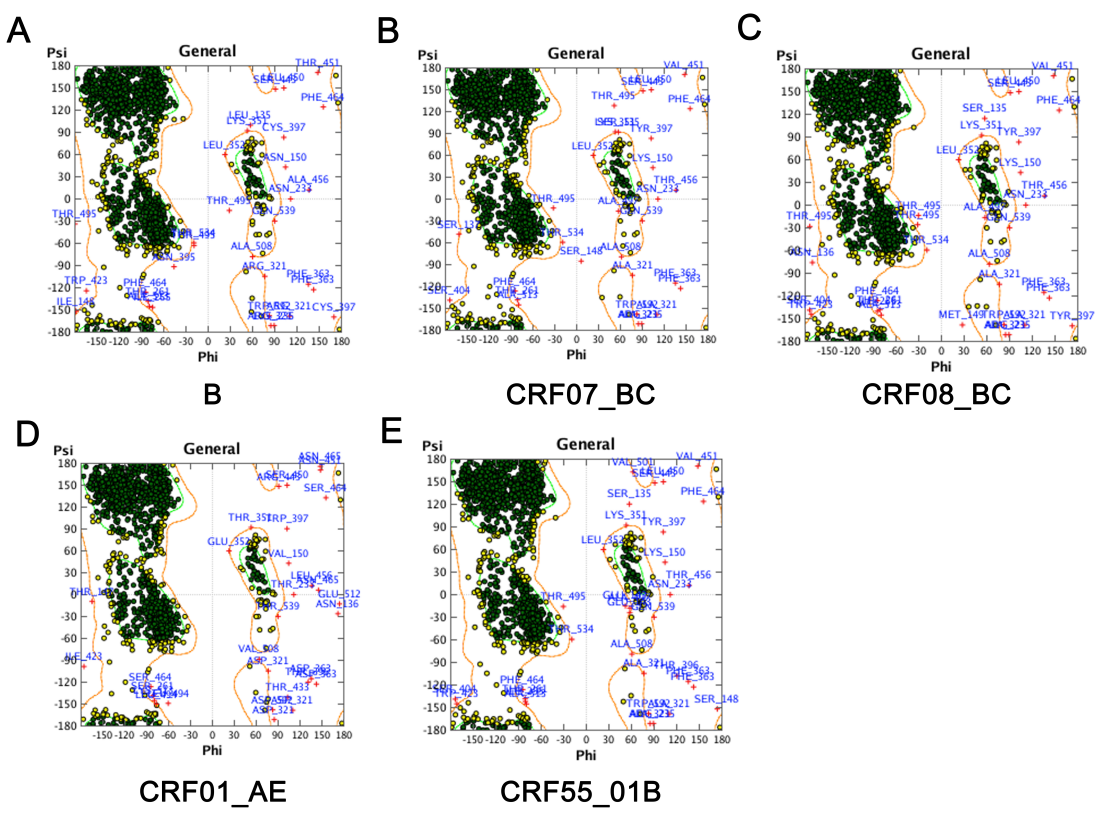


**Supplementary Figure 8.Ramachandran plot analysis of the optimal model-1 structures of proteins from gp140 of different HIV subtypes.**

**
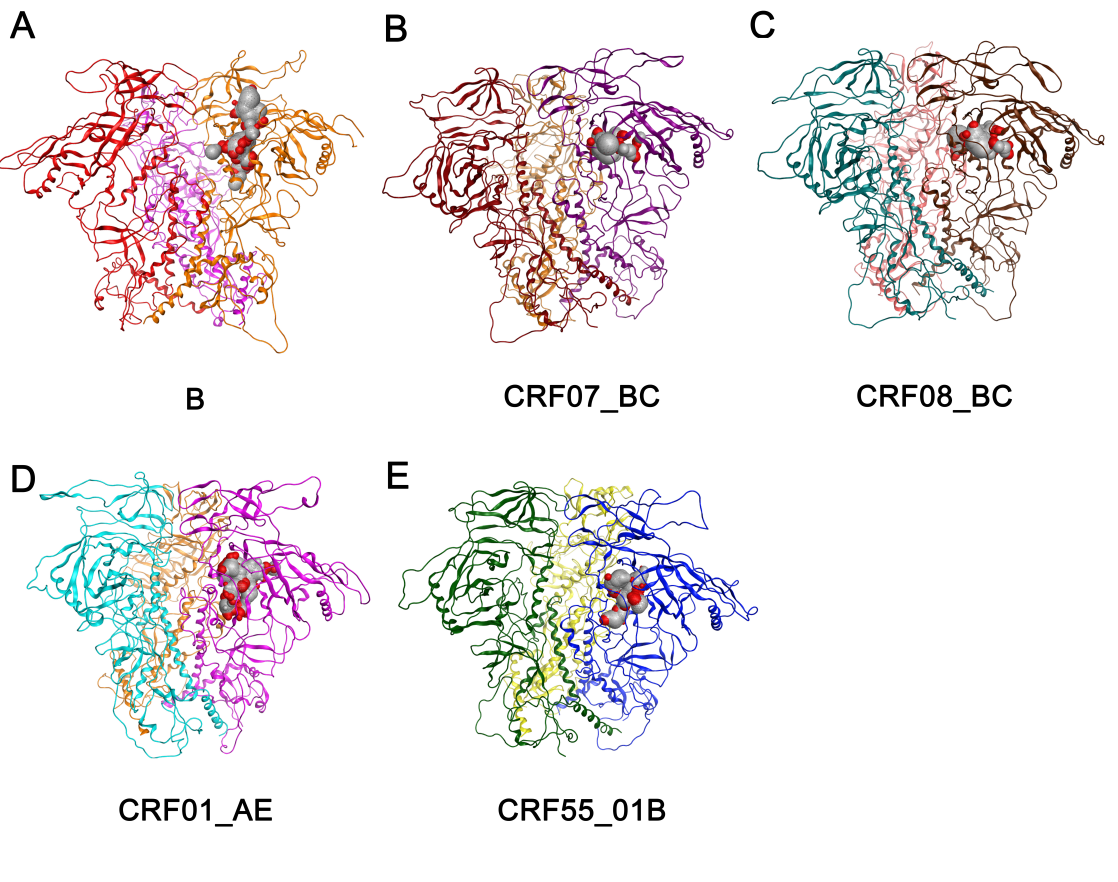
**

**Supplementary Figure 9.Prediction of the binding sites between the gp140 structures of different subtypes of HIV and small molecules.**

**
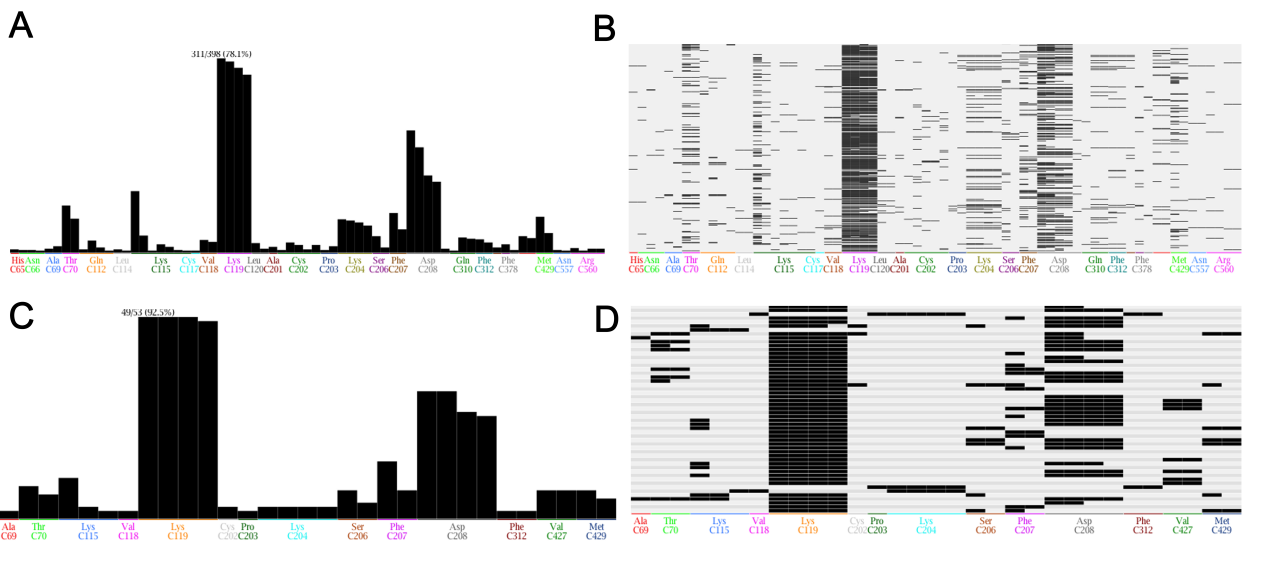
**

**Supplementary Figure 10.The frequency of amino acid sites involved in binding to small molecule compounds in virtual screening.** (A) Amino acid sites involved in binding to 399 compounds; (B) The frequency of amino acid sites involved in binding to small molecule compounds.(C) Amino acid sites involved in binding to 53 compounds;(D) The frequency of amino acid sites involved in binding to small molecule compounds.

**
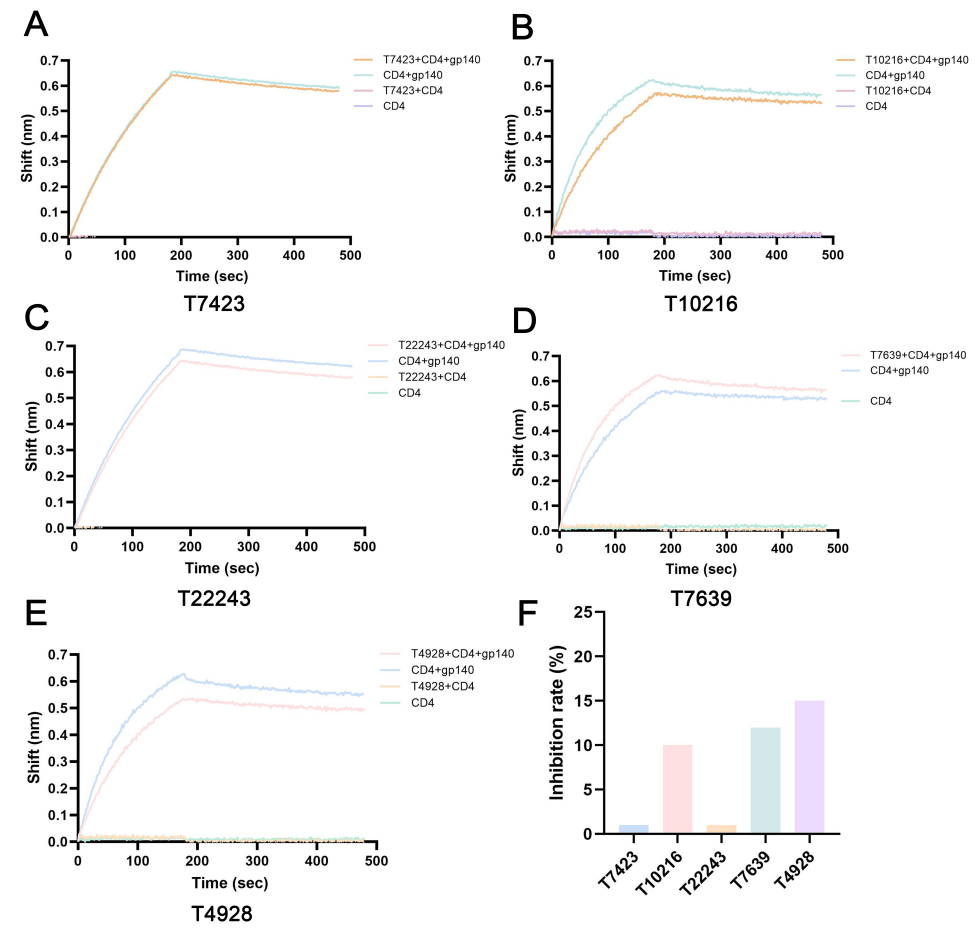
**

**Supplementary Figure 11. BLI detects the effect of small molecule inhibitors on inhibiting the binding of gp140 of HIV subtype CRF55_01B to CD4.** (A) T7423, (B) T10216, (C) T22243, (D) T7639, (E) T4928, (F) Inhibition Rates of Compounds on HIV Adhesion Process.


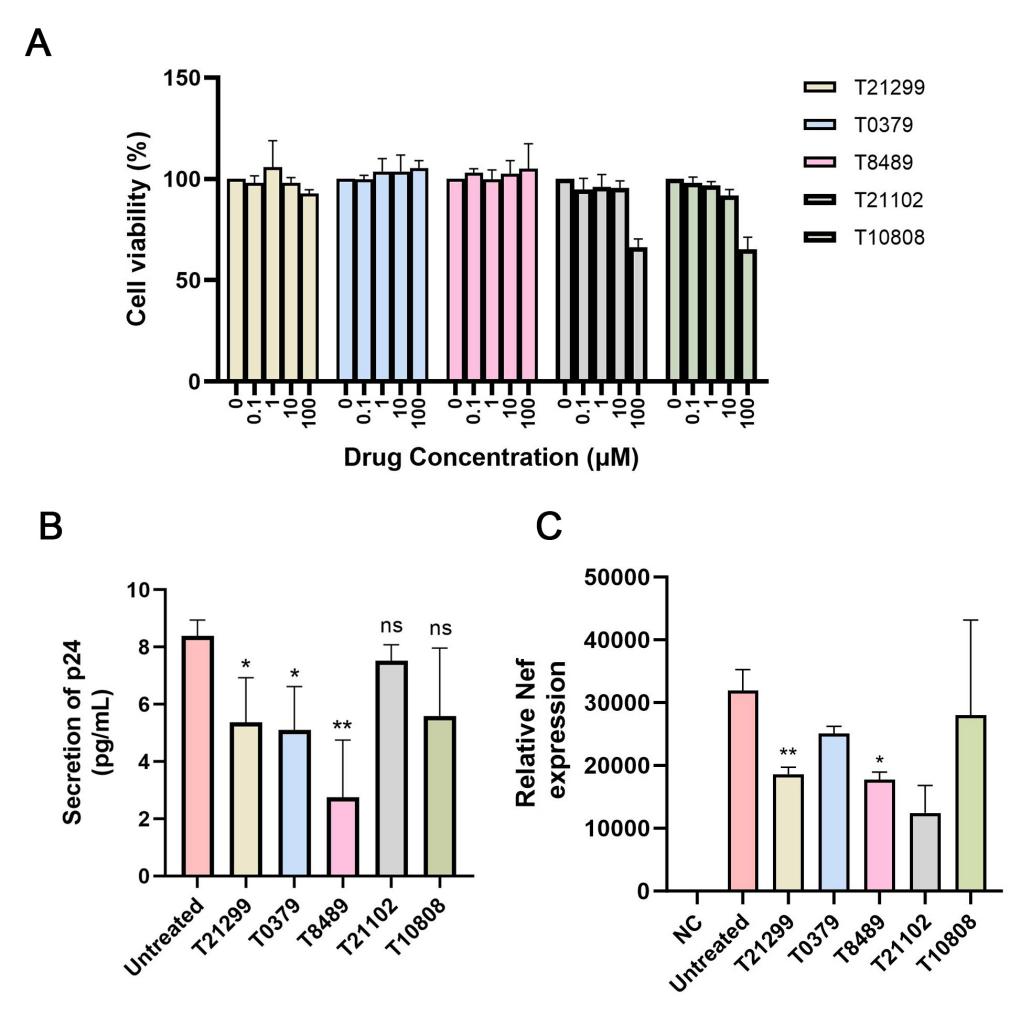


**Supplementary Figure 12. Inhibitory effects of small molecule compounds on HIV infection in TZM-bl cells.** (A) CCK-8 assay detecting the cytotoxicity of 0.1-100 μM compounds on TZM-bl cells after 48h treatment, (B) ELISA measuring p24 levels in the cell culture supernatant at 48h after infection with HIV pseudovirus pre-incubated with 10 μM compounds for 2 hours, (C) qRT-PCR detecting HIV-1 nef gene expression in TZM-bl cells at 48h after infection with HIV pseudovirus pre-incubated with 10 μM compounds for 2 hours. The assays were performed three times in triplicate. The data presented are mean values ± the standard deviations. NC:negative control. * *p＜ 0.05,* ** *p＜ 0.01, ns:Not Significant.*


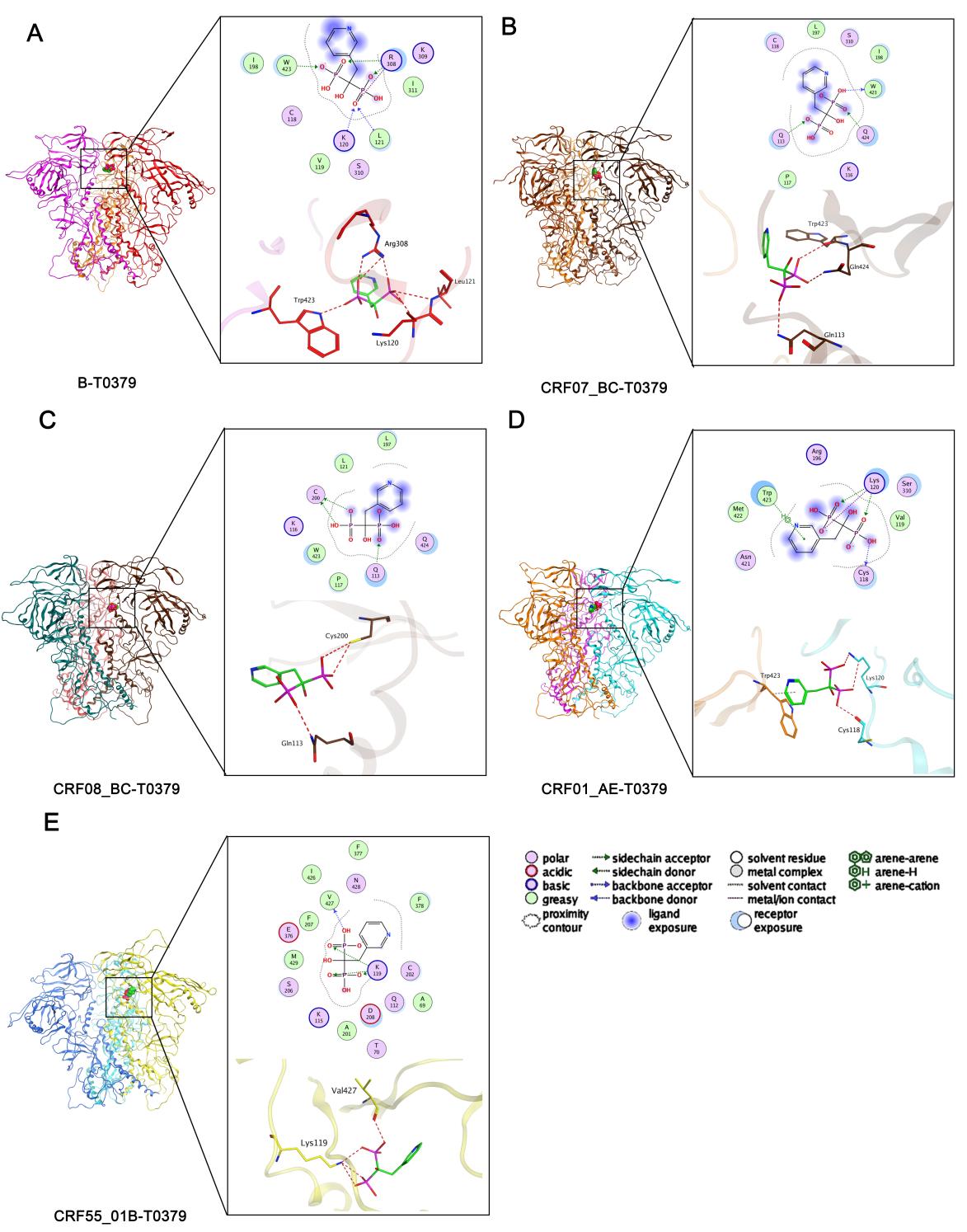


**Supplementary Figure 13. Docking of different subtypes of HIV gp140 with the binding site of the small molecule compound T0379.**


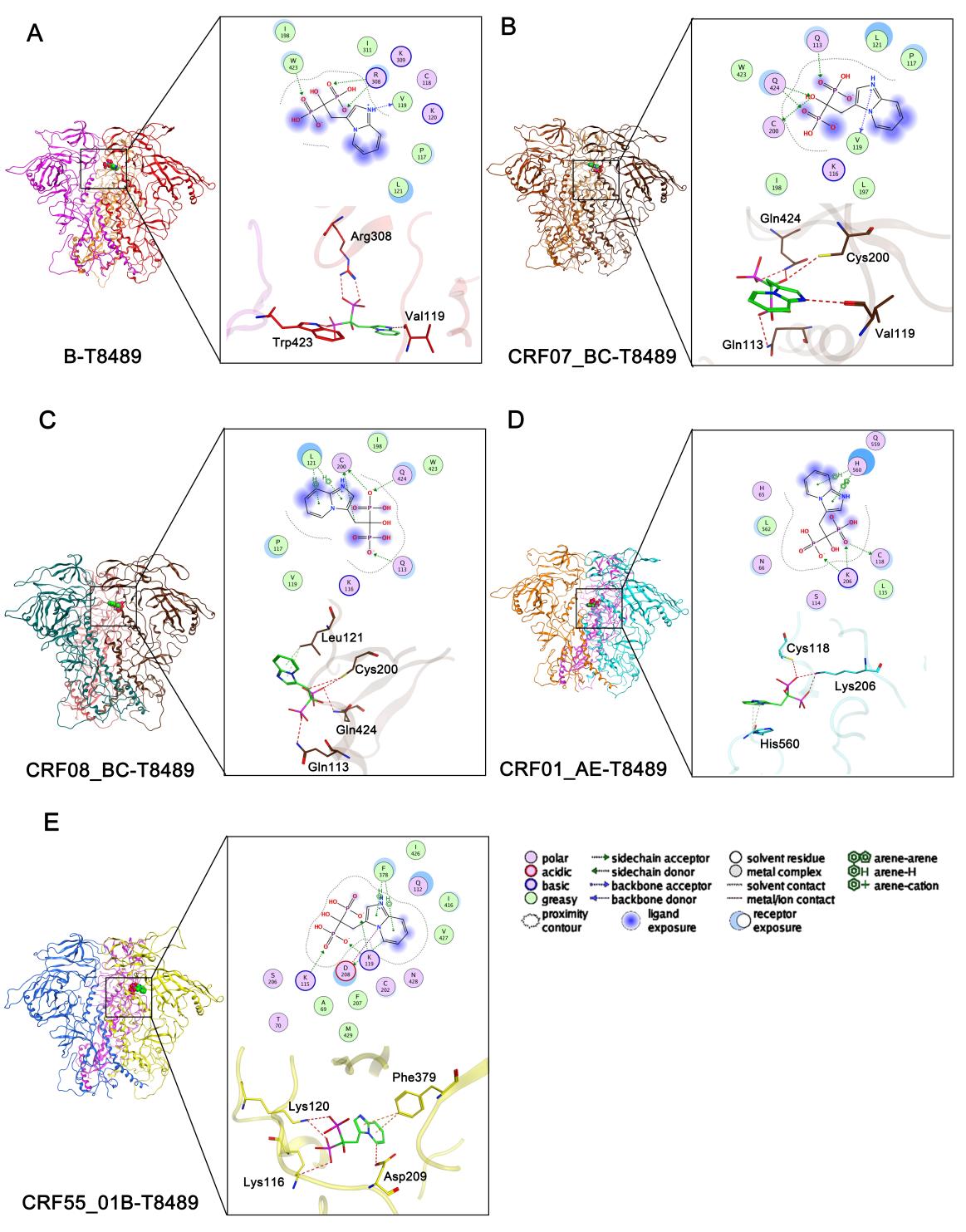


**Supplementary Figure 14. Docking of different subtypes of HIV gp140 with the binding site of the small molecule compound T8489.**


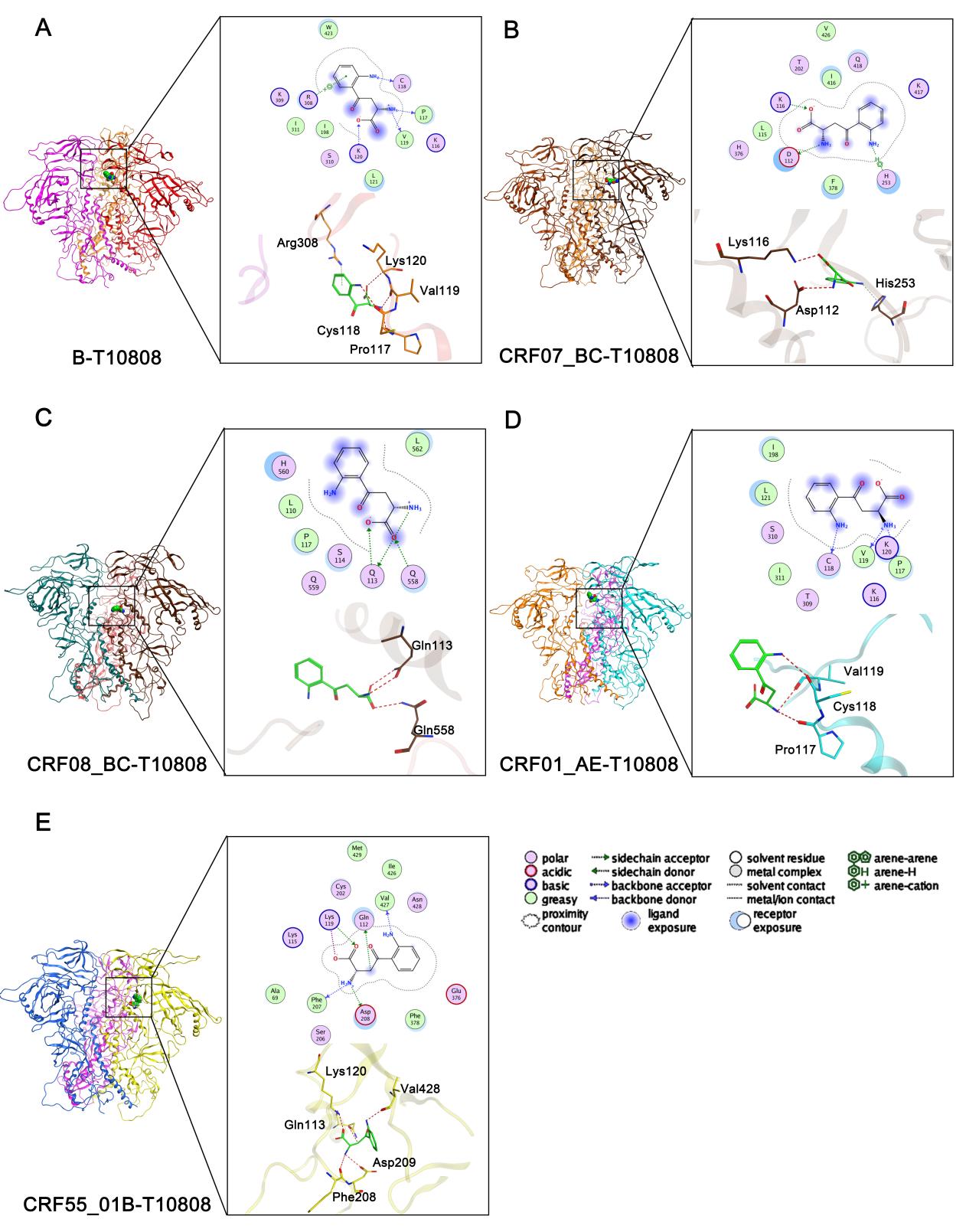


**Supplementary Figure 15. Docking of different subtypes of HIV gp140 with the binding site of the small molecule compound T10808.**


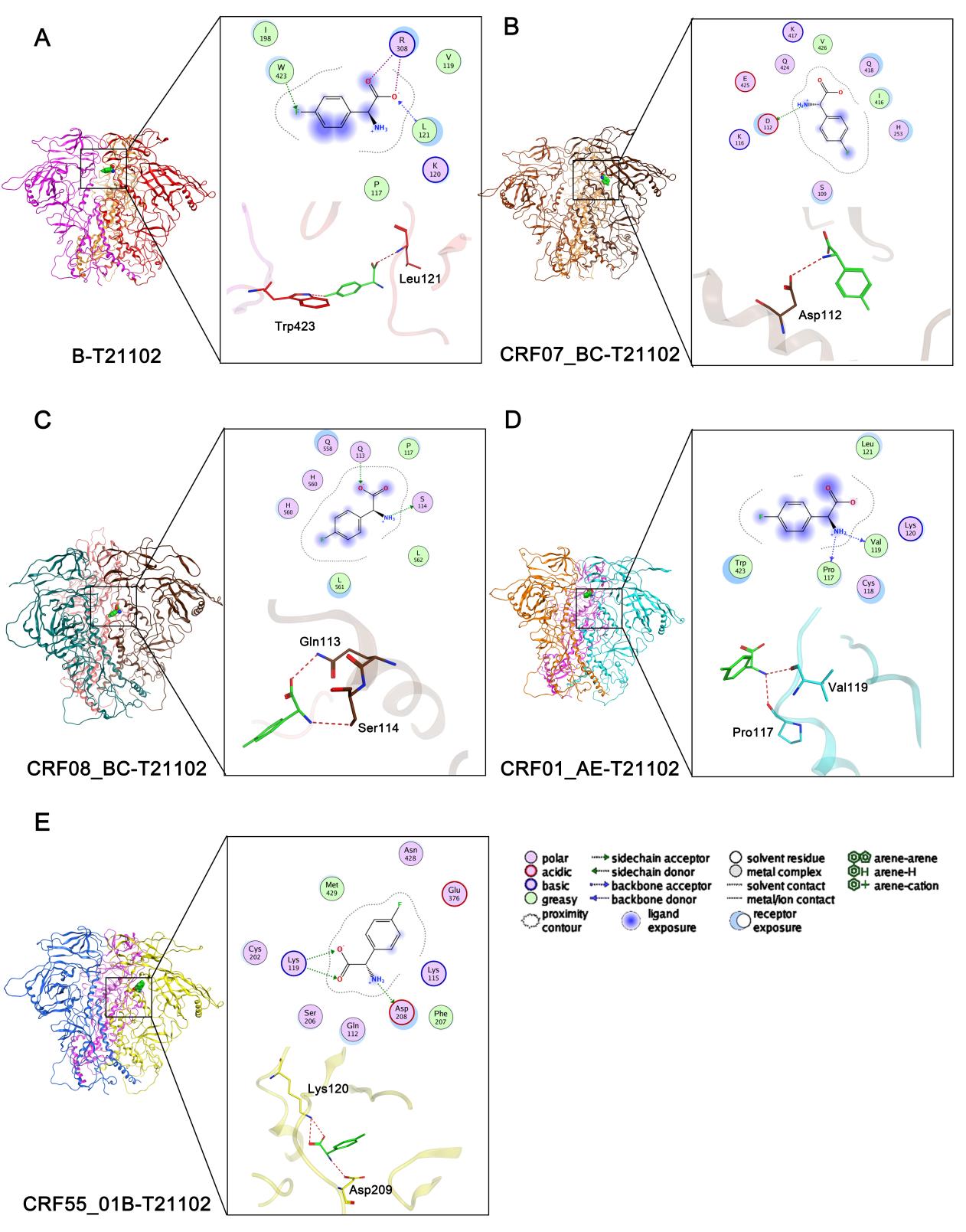


**Supplementary Figure 16. Docking of different subtypes of HIV gp140 with the binding site of the small molecule compound T21102.**


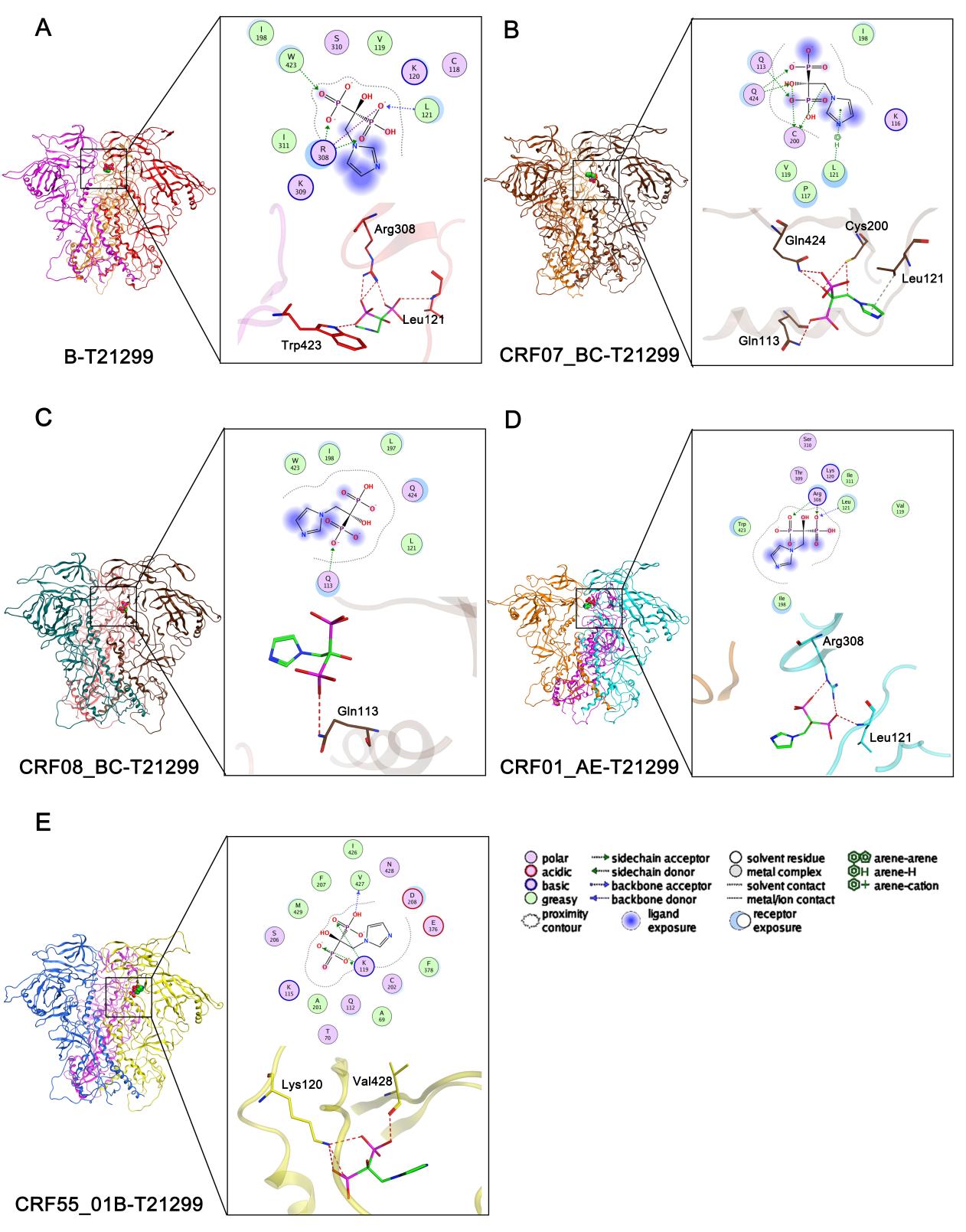


**Supplementary Figure 17. Docking of different subtypes of HIV gp140 with the binding site of the small molecule compound T21299.**
